# *STX17-DT* facilitates axitinib resistance in renal cell carcinoma by inhibiting mitochondrial ROS accumulation and ferroptosis

**DOI:** 10.1038/s41419-025-07456-9

**Published:** 2025-02-23

**Authors:** Yihui Pan, Shuang Liu, Guannan Shu, Minyu Chen, Liangmin Fu, Cheng Chen, Yimeng Chen, Qianfeng Zhuang, Dong Xue, Xiaozhou He

**Affiliations:** 1 Department of Urology, the Third Affiliated Hospital of Soochow University, Changzhou, China; 2 Department of Oncology, the Third Affiliated Hospital of Soochow University, Changzhou, China; 3 Department of Urology, Guangzhou Women and Children’s Medical Center, Guangzhou Medical University, Guangdong Provincial Clinical Research Center for Child Health, Guangzhou Medical University, Guangzhou, Guangdong China; 4 Department of Pediatric Surgery, Guangzhou Institute of Pediatrics, Guangdong Provincial Key Laboratory of Research in Structural Birth Defect Disease, Guangzhou Women and Children’s Medical Center, Guangzhou Medical University, Guangzhou, Guangdong China; 5 Department of Urology, the First Affiliated Hospital, Sun Yat-sen University, Guangzhou, China; 6 Department of Urology, the Second Xiangya Hospital, Central South University, Changsha, Hunan China; 7 Uro-Oncology Institute of Central South University, Changsha, Hunan China

**Keywords:** Cancer therapeutic resistance, Renal cell carcinoma

## Abstract

Axitinib resistance remains a serious challenge in the treatment of advanced renal cell carcinoma (RCC), and the underlying mechanisms are not fully understood. Here, we constructed an in vivo axitinib-resistant RCC model and identified the long non-coding RNA *STX17-DT* as a driver of therapy resistance in RCC. The expression of *STX17-DT* was significantly elevated in axitinib-resistant RCC cells and correlated with poorer prognosis in RCC patients. Elevated levels of *STX17-DT* contributed to the development of resistance to axitinib both in vitro and in vivo. Mechanistically, *STX17-DT* modulated the stability of *IFI6* mRNA by recruiting and binding to hnRNPA1, leading to decreased accumulation of mitochondrial reactive oxygen species (ROS) and attenuated ferroptosis. Meanwhile, *STX17-DT* was packaged into extracellular vesicles through hnRNPA1, thus transmitting axitinib resistance to other cells. Compared with axitinib monotherapy, combined treatment of axitinib and *STX17-DT-*targeted in vivo siRNA demonstrated enhanced therapeutic efficacy. These findings indicate a novel molecular mechanism of axitinib resistance in RCC and suggest that *STX17-DT* may serve as a prognostic indicator and potential therapeutic target to overcome resistance to targeted therapy.

## Introduction

Renal cell carcinoma (RCC) originates from the epithelial cells of the renal tubules and is a common tumor of the urinary system with an increased incidence in recent years [1]. Almost 70% of RCCs are clear cell renal cell carcinomas (ccRCCs) in terms of pathology [2]. Approximately 30% of patients develop distant metastases at initial diagnosis, ultimately leading to a poor prognosis [3, 4]. Axitinib is a multitarget tyrosine kinase inhibitor that mainly affects the VEGFR and PDGFRβ signaling pathways. In recent years, the combination of axitinib and pembrolizumab has been recommended by international guidelines as a first-line treatment for advanced metastatic RCC, resulting in significantly improved outcomes [5, 6]. However, a large proportion of patients with metastatic RCC eventually develop resistance to axitinib, severely limiting its clinical application [7]. Although some previous studies have identified efficacy biomarkers for axitinib treatment, the mechanisms underlying axitinib resistance have not been fully elucidated.

Ferroptosis is a non-apoptotic cell death mechanism characterized by iron-dependent lipid peroxidation [8, 9]. The oncogenic RAS-selective lethal small molecule erastin was initially discovered to induce ferroptosis [10]. Ferroptosis is characterized by distinct morphological features such as decreased mitochondrial volume, rupture of the mitochondrial outer membrane, reduction or absence of mitochondrial cristae, and maintenance of normal nuclear concentration, setting it apart from other types of cell death [11, 12]. Another important feature of ferroptosis is the imbalance of redox status and the increase in intracellular reactive oxygen species levels [13]. Dysregulation of ferroptosis has been shown to be associated with cancer, neurodegenerative diseases, tissue damage, inflammation, and infection [13, 14]. Since the development of apoptosis resistance in cancer cells, non-apoptotic cell death mechanisms like ferroptosis are promising approaches for combatting drug-resistant cancers [15, 16]. Studies have revealed that cells resistant to traditional treatments exhibit increased sensitivity to ferroptosis. The activation of ferroptosis may be a potential strategy to overcome traditional resistance to cancer treatment [17, 18].

The human genome contains a significant number of long non-coding RNAs (lncRNAs) that serve diverse functions in gene regulation, cell biology and human diseases [19]. LncRNAs are dysregulated in various malignant tumors and interact with multiple RNAs and proteins to influence drug resistance [20, 21]. LncRNAs regulate drug resistance in cancer through multiple molecular mechanisms, including multidrug efflux, inhibition of cell apoptosis, DNA damage response, epigenetic alterations, and function as competitive endogenous RNAs [22, 23]. Moreover, lncRNAs with specific sequences can be packaged into extracellular vesicles (EVs) and transferred to neighboring tumor cells, leading to the spread of drug resistance [24]. LncRNAs that induce drug resistance may serve as valuable targets for the development of personalized therapeutics aimed at overcoming therapy resistance, thus offering a new avenue for innovative and tailored treatment strategies. However, the role of lncRNAs in axitinib resistance still needs further exploration.

In this study, we constructed an in vivo model of axitinib-resistant RCC cell lines and first identified a novel axitinib resistance-associated lncRNA *STX17-DT*. A series of in vitro and in vivo experiments were performed to validate the effect of *STX17-DT* on axitinib resistance. Mechanistically, *STX17-DT* promoted axitinib resistance through direct binding to hnRNPA1, thereby stabilizing *IFI6* mRNA, leading to ROS clearance and ferroptosis suppression. Overall, this study provides new insights into the molecular-targeted therapy and potential targets for reversing axitinib resistance in advanced RCC.

## Methods

### Cells and cell culture

786-O and 769-P cells were purchased from the Cell Bank of the Chinese Academy of Sciences and maintained in RPMI-1640 medium (Gibco) supplemented with 10% FBS (PAN-Seratech) and 1% penicillin-streptomycin (Biosharp) at 37 °C with 5% CO_2_. Prior to experimentation, vendor-conducted short-tandem repeat profiling was performed on all the cell lines. Furthermore, regular testing for Mycoplasma infection was also carried out on all the cell lines.

### Patients and specimens

All human ccRCC tissue samples were obtained from the Department of Urology, the Third Affiliated Hospital of Soochow University (Changzhou, China). The protocol was approved by the Medical Ethics Committee of Third Affiliated Hospital of Soochow University. Written informed consent was obtained from all participants and their surrogates for the use of human ccRCC tissue specimens samples. All patients underwent nephrectomy prior to targeted therapy and were administered axitinib as second-line treatment following the failure of sunitinib. All the tested tissues were primary tumors. Patients were excluded if their laboratory examination information and follow-up data were incomplete, or if their RNA contents were less than 10 ng/μL. A total of 42 ccRCC patients who received axitinib treatment between 2019 and 2023 were ultimately recruited from the Third Affiliated Hospital of Soochow University. The detailed clinical characteristics are provided in Supplementary Tables 1, 2. Tumor specimens used for the PDX models were obtained in 2023 from the Third Affiliated Hospital of Soochow University. The detailed clinical characteristics are provided in Supplementary Table 3. Progressive disease is characterized by the emergence of new lesions or a 20% increase in the combined diameters of the target lesions. Progression-free survival (PFS) is defined as the duration from the initiation of axitinib therapy to the first detection of radiological evidence indicating disease progression.

### Animal studies

All animal experiments in this study were approved and conducted in accordance with the guidelines of Institutional Animal Care and Use Committee of Sun Yat-sen University Cancer Center. Four- to five-week-old male BALB/c-Nu mice and NCG (NOD/ShiLtJGpt-Prkdcem26Cd52Il2rgem26Cd22/Gpt) mice were purchased from GemPharmatech (China) and fed under specific pathogen-free (SPF) conditions. The mouse models had mice randomly assigned to groups.

For the generation of axitinib-resistant RCC cell models, 4-week-old male BALB/c-Nu mice were housed under SPF conditions. 786-O and 769-P cells were injected subcutaneously at a concentration of 5 × 10^6^ cells/100 μL. Two weeks later, when the mean tumor volume reached 200 mm^3^, the first-passage mice were treated with axitinib (10 mg/kg/day) or vehicle orally for 6 weeks. Axitinib was procured from MCE and dissolved in a solution containing 10% DMSO and 90% corn oil. After six weeks, the tumors were excised from euthanized mice and sectioned into 1 mm^3^ blocks. These tumor blocks were subsequently transplanted into the next passage of mice, which were subjected to either axitinib treatment or vehicle treatment. After the sacrifice of third-passage mice, the tumors were digested in ACCUMAX™ solution at 37 °C for 30 min with continuous shaking. The cell suspension was centrifuged at 300 × *g* and the supernatant was plated in a 6-well plate at a concentration of 5 × 10^5^ cells/well. Fibroblasts were removed via the reduplicative adherence method. Axitinib-resistant RCC cells were named 786-O-R and 769-P-R.

For the subcutaneous xenograft tumor model, preprocessed 786-O cells were injected into BALB/c-Nu mice at a concentration of 5 × 10^6^ cells /100 μL. Tumor volume was measured weekly. When the tumor volume reached 200 mm^3^, the mice were treated with axitinib (10 mg/kg/day) or vehicle orally for 6 weeks. For the orthotopic xenograft tumor model, all the mice were anesthetized with 1% pentobarbital (50 mg/kg body weight) in PBS. Preprocessed 786-O cells were injected into the renal subcapsule of BALB/c-Nu mice at a concentration of 10^6^ cells/100 μL. Two weeks later, all the mice were treated with axitinib (10 mg/kg/day) orally. Six weeks after the drug treatment, all mice were euthanized.

For the extracellular vesicle (EV) studies, we constructed a subcutaneous xenograft tumor model as described above. Two weeks later, all the mice were injected with EVs (100 μg/week) from CM of treated 786-O cells or PBS intratumourally and treated with axitinib (10 mg/kg/day) orally. Six weeks after drug treatment, all the mice were euthanized.

For the ccRCC PDX models, NCG mice were subcutaneously transplanted with fresh human ccRCC tissue fragments on their flanks. When the tumor volume reached 100 mm^3^, the mice were euthanized. The tumors were cut into 1 mm^3^ pieces and transplanted into second-generation mice. After three generations, a stable ccRCC PDX model was obtained. We established five ccRCC PDX models and selected two models with elevated IC_50_ values for axitinib for further experimentation.

For the in vivo si-*STX17-DT* experiment, NCG mice were first anesthetized with 1% pentobarbital (50 mg/kg body weight) in PBS. A piece of PDX tumor (1 mm^3^) was orthotopically transplanted into the right renal subcapsule and fixed with 3 M Vetbond Tissue Adhesive. Two weeks later, all the mice were treated with axitinib (10 mg/kg/day) orally and injected with in vivo si-*STX17-DT* (10 nmol/week, RiboBio, China) intratumorally for 6 weeks. Six weeks after drug treatment, all the mice were euthanized.

### Half inhibitory concentration assay

A total of 3000 cells were seeded in 96-well plates and treated with 1, 10, 100, 1000, 10,000, and 1,00,000 nM axitinib for 48 h. Cell viability was measured using the CCK-8 assay (MCE). The cell viability was measured by OD450 with a microplate reader. The half-inhibitory concentration (IC_50_) was calculated using Graph Pad Prism 9 (Graph Pad Software Inc., San Diego, CA, USA), and a dose-response curve was plotted via the equation log (inhibitor) vs. response-variable slope.

### Calcein-AM assay

A total of 3000 cells were inoculated in 96-well plates and treated with 5 μM axitinib for 48 h. Cell death was measured using the Calcein-AM/PI Cell Viability/Cytotoxicity Assay Kit (Biosharp) under a fluorescence microscope. Live cells are stained green, whereas dead cells are stained red.

### Colony formation

The logarithm of the cell growth phase of 786-O and 769-P cells was taken as 500/well, and the cells were inoculated in 12-well plates with 2 mL of growth media and incubated at 37 °C for 14 days. After that, the cells were washed with PBS and fixed with 4% paraformaldehyde for 15 min. Following PBS washed, cells were stained with 0.1% crystal violet for 15 min. The staining solution was then removed and the plates were washed three times with running water to remove the excess stain and allowed to dry at room temperature. The number of colonies (>50 cells) was counted under the microscope.

### RNA pull-down, silver staining, and mass spectrometry analysis

An RNA pulldown assay was conducted using the Pierce™ Magnetic RNA-Protein Pull-Down Kit in accordance with the manufacturer’s instructions (Thermo Fisher Scientific). Biotin-labeled *STX17-DT* probes and antisense probes were synthesized by in vitro transcription utilizing T7 RNA polymerase and Biotin RNA Labeling Mix (Invitrogen). The pulldown proteins were subjected to SDS-PAGE gel electrophoresis, and the gel was stained with a Fast Silver Stain Kit (Beyotime). Mass spectrometry analysis was performed at the Beijing Genomics Institution (BGI).

### RNA immunoprecipitation

RNA immunoprecipitation was conducted with an EZ-Magna RIP Kit (Millipore, USA) according to the manufacturer’s instructions. Briefly, 10^7^ ccRCC cells were harvested and exposed to RIP lysis buffer supplemented with protease and RNase inhibitors. Supernatants were isolated and incubated with anti-hnRNPA1 antibody overnight at 4 °C. After that they were mixed with the protein A/G beads. RNA was then extracted from the eluate, and the enrichment of *STX17-DT* was assessed via qRT-PCR. Normal IgG served as the negative control.

### Electrophoretic mobility shift assay

EMSA experiments were conducted using a LightShift Chemiluminescent EMSA Kit (Thermo Scientific, USA). Biotin-labeled *STX17-DT* was synthesized through in vitro transcription using T7 RNA polymerase and Biotin RNA Labeling Mix (Beyotime, China). Biotin-labeled *STX17-DT* was then incubated with flag-tagged hnRNPA1 at room temperature for 15 min in EMSA binding buffer. Following separation by PAGE, signal detection was carried out using the Chemiluminescent Nucleic Acid Detection Module (Thermo Scientific Pierce, USA).

### Fluorescent in situ hybridization

The Cy3-conjugated *STX17-DT* probe was developed by RiboBio (Guangzhou, China). RNA FISH was conducted using the fluorescent in situ hybridization kit from RiboBio, following the manufacturer’s guidelines. Briefly, RCC cells were seeded in confocal dishes, fixed with 4% paraformaldehyde at room temperature for 10 min, permeabilized with 0.5% Triton in PBS, and subjected to prehybridization at 37 °C for 30 min. Subsequently, cells were incubated with the probes (5 μM) overnight at 37 °C. Cy3-labeled *U6* and *18S* probes supplied by RiboBio served as controls for nuclear and cytoplasmic RNA, respectively. The samples were counterstained with DAPI and examined using an FV1000 confocal laser microscope (Olympus, Japan).

### Detection of ferroptosis-related markers

Mitochondrial superoxide was detected using a Mitochondrial Superoxide Detection Kit (Dojindo) according to the manufacturer’s instructions. Mitochondrial lipid peroxide was detected using a Mitochondrial Lipid Peroxide Detection Kit (Dojindo). GSH was detected using a GSH Quantification Kit (Dojindo) according to the manufacturer’s instructions. The detection of ferrous ions (Fe^2+^) was performed using the Mitochondrial Iron Detection Kit according to the manufacturer’s instructions.

### Bioinformatics

Differential expression analysis of the mRNAs and lncRNAs was conducted with R software v4.3.2 using the tidyverse, DESeq2, and pheatmap packages. Protein coding potential analysis of *STX17-DT* was conducted using the LNCipedia (https://lncipedia.org/). The subcellular localization of *STX17-DT* was predicted using lncLocator (http://www.csbio.sjtu.edu.cn/bioinf/lncLocator/). Gene set enrichment analysis was conducted to compare the enriched pathways between different groups. The predicted secondary structure of *STX17-DT* was downloaded from the RNAfold web server (http://rna.tbi.univie.ac.at/cgi-bin/RNAWebSuite/RNAfold.cgi).

### RNA-sequencing and analysis of lncRNAs

Axitinib-resistant and axitinib-sensitive RCC cells were stored in TRIzol reagent (Invitrogen) and rapidly frozen in liquid nitrogen. Before sequencing, ribosomal RNA (rRNA) was removed from the total RNA. RNA sequencing was conducted by RiboBio (Azenta, China) based on an Illumina sequencing platform using the PE150 and SE50 methods. The raw counts were normalized to fragments per kilobase million (FPKM).

### Dual-luciferase assays

The promoter and 3’-UTR activities of *IFI6* were analyzed by dual-luciferase reporter assays. *IFI6*-3’-UTR was inserted downstream of the luciferase gene in the psiCHECK2 plasmids, while *IFI6*-promoter was inserted upstream of the luciferase gene in the pGL3 plasmids. The cells were seeded in 6-well plates and transfected with the constructed psiCHECK2 or pGL3 plasmids. The empty psiCHECK2 or pGL3 vector was used as a negative control. Luciferase activity was measured using a Dual Luciferase Reporter Gene Assay Kit (YEASEN, China) and a GloMax20/20 Luminometer (Promega, USA). Normalized firefly activity was obtained as the ratio of firefly to Renilla luciferase signals.

### Extracellular vesicle (EV) experiments

RCC cells were grown in cell medium supplemented with 10% EV-depleted FBS (SBI) for 72 h before the isolation of the EVs. The supernatant was harvested and centrifuged at 300 × *g* for 10 min followed by centrifugation at 2000 × *g* for 10 min, and then at 10,000 × *g* for an additional 10 min. Subsequently, the supernatant was filtered through a 0.22 μm filter, followed by two rounds of centrifugation at 1,20,000 × *g* for 80 min each. The resulting pellets were resuspended in PBS for subsequent experiments.

To isolate EVs from plasma, an equal volume of serum was collected and diluted with PBS. Total Exosome Isolation (from plasma) Kit (Invitrogen) was then employed following the manufacturer’s instructions to isolate EVs from plasma.

EV morphology was examined by JEM1200-EX transmission electron microscope (TEM) (JEOL, Japan). The size of EVs was detected by Nanosight ns300 (Malvern Instruments Ltd., Malvern, UK). EV-specific markers were investigated using western blotting. The specific primary antibodies applied in this study were listed in Supplementary Table 6.

For qRT–PCR analyses of the EVs, synthesized *λ* polyA^+^ RNA (Takara, China) was utilized as the external reference. Briefly, lncRNAs were normalized to *λ* polyA^+^ RNAs per million EV particles. Specifically, 1.8 × 10^8^ *λ* polyA^+^ RNA was added to the EV suspension before RNA extraction. The number of EV particles was quantified using the NTA 3.0 software. RNA extraction was carried out using the Total Exosome RNA and Protein Isolation Kit (Invitrogen), following the manufacturer’s instructions.

### Other experiments

The detailed procedures for other experiments, such as western blotting, RNA extraction, qRT–PCR, RNA interference, and immunohistochemical staining, have been described in previous studies [24].

### Statistical analyses

Statistical analyses were performed using SPSS version 25.0 or GraphPad Prism 9.0 software. All in vitro experiments were conducted in three independent biological replicates. The data were analyzed for normality before comparison. For comparisons between two groups, statistical significance was determined by two-tailed Student’s *t*-test. For multiple comparisons, one-way ANOVA with Tukey’s post hoc test was employed. Kaplan–Meier survival curves were generated for survival analyses, and log-rank tests were utilized. Correlation analyses were performed using Pearson’s correlation (for continuous variables) or Spearman’s correlation (for discontinuous variables). The data are presented as mean ± standard deviation (SD). A *P* value less than 0.05 was considered significant. Statistical significance was shown as *P < 0.05, **P < 0.01 or ***P < 0.001.

## Results

### Construction of axitinib-resistant cell lines in vivo

**Fig. 1:**
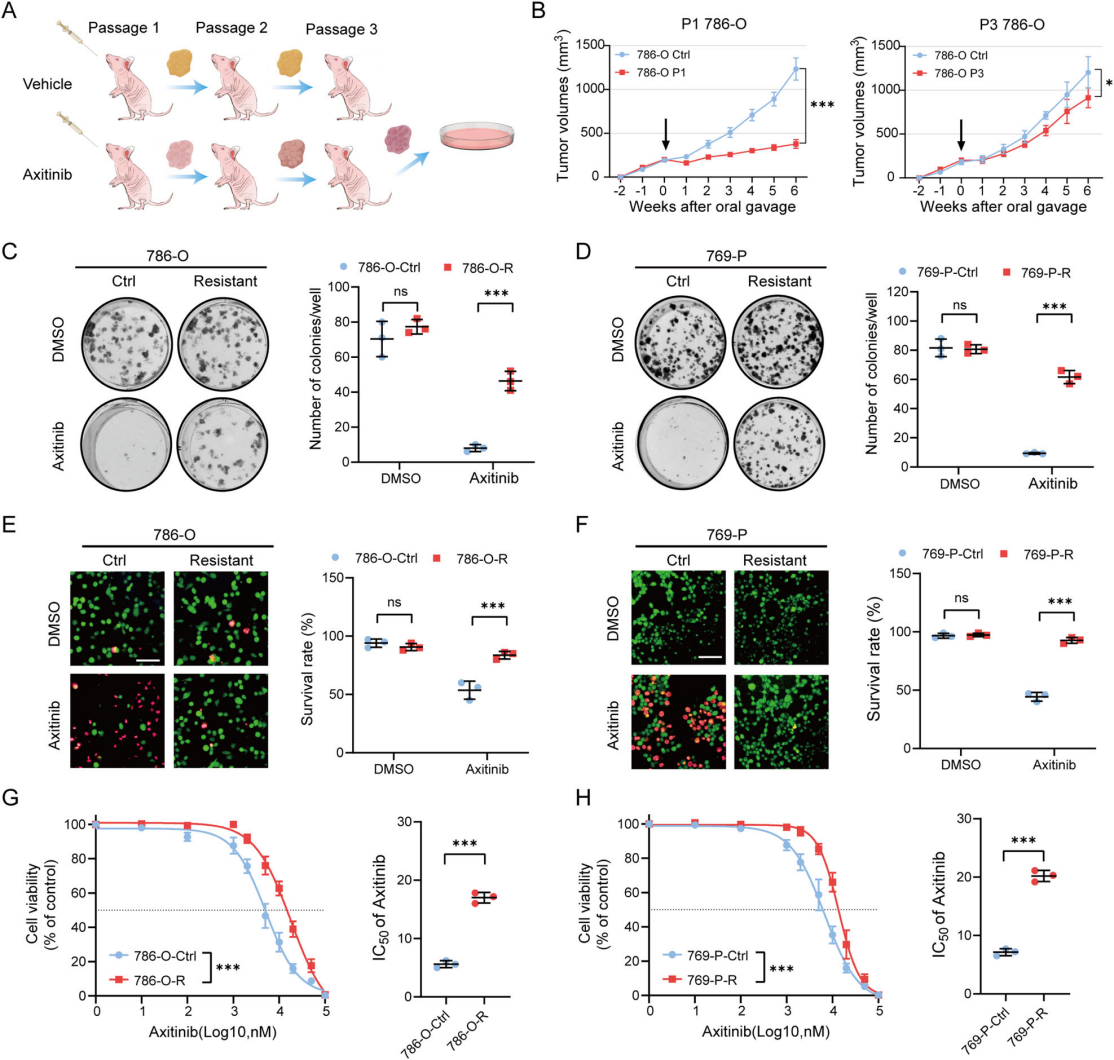
Construction of axitinib-resistant cell lines in vivo. **A** Schematic illustration depicting the modeling process for the acquisition of axitinib-resistant RCC cells. **B** The construction of axitinib-resistant RCC cells through three successive passages. The arrow indicates the start of treatment. **C**, **D** Colony formation assays of resistant and control RCC cells after treated with axitinib. **E**, **F** Representative images of Calcein-AM/PI staining of resistant and control RCC cells after treated with axitinib. Scale bar, 100 μm. **G**, **H** Cell viability curves and IC_50_ values of resistant and control RCC cells. The data are shown as mean ± SD (error bars).

We established axitinib-resistant cell lines by injecting 786-O and 769-P cells into nude mice and performing axitinib treatment in vivo. The cells gradually acquired increased resistance to axitinib therapy over the passages (Figs. 1A, B and S1A, S1B). We carried out a series of in vitro experiments to determine the resistance of 786-O-R and 769-P-R cells. As expected, compared with parental control cells, 786-O-R and 769-P-R cells exhibited stronger clone formation abilities, higher cell liabilities, and higher IC_50_ values when treated with axitinib (Fig. 1C–H).

### *STX17-DT* was upregulated in axitinib-resistant RCC cells and associated with poor outcomes in RCC patients

**Fig. 2:**
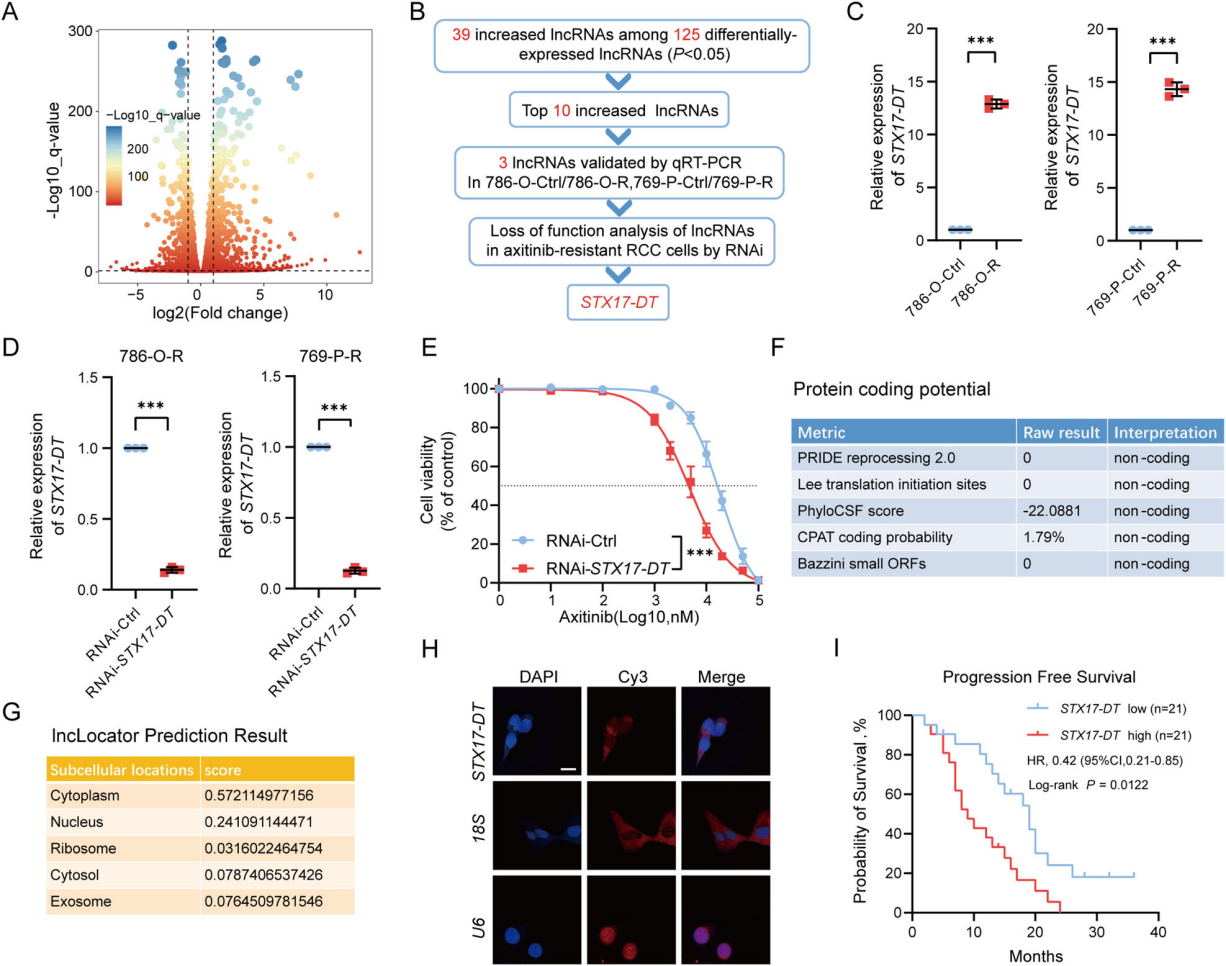
*STX17-DT* was upregulated in axitinib-resistant cells and associated with poor outcomes in ccRCC patients. **A** Differentially expressed genes in sensitive and resistant 786-O cells according to transcriptome sequencing. **B** Flowchart of the screening strategy for genes related to axitinib resistance. **C** Relative expression of *STX17-DT* was validated by qRT‒PCR in sensitive and resistant RCC cells. **D** Relative expression of *STX17-DT* was validated by qRT‒PCR after silencing. **E** Cell viability assay in *STX17-DT*-silenced RCC cells. **F** The coding potential of *STX17-DT* was analyzed using LNCipedia (https://lncipedia.org/). **G** Subcellular localization of *STX17-DT* was predicted by lncLocator (http://www.csbio.sjtu.edu.cn/bioinf/lncLocator/). **H** Representative RNA FISH images of *STX17-DT*, *U6,* and *18S* in 786-O cells. *U6* is the positive control for the nucleus, and *18S* is the positive control for the cytoplasm. Scale bar, 20 μm. **I** Kaplan‒Meier survival analysis of the PFS of ccRCC patients with high vs. low *STX17-DT* expression in the SCU cohort. The data are shown as mean ± SD (error bars).

To elucidate the mechanism of axitinib resistance in RCC, transcriptomic differences between the drug-sensitive and drug-resistant cell lines were investigated (Fig. 2A). The differentially expressed lncRNAs are shown in the heatmap (Fig. S2A). We detected the top ten most significantly upregulated lncRNAs in axitinib-resistant cells through qRT-PCR, and three of them were validated (Figs. 2B, C and S2B, S2C). To further screen the lncRNAs involved in axitinib resistance, we then performed in vitro IC_50_ assays and found that silencing *STX17-DT* reduced the axitinib resistance of RCC cell lines most obviously (Figs. 2D, E and S2D–S2G). We then focused on *STX17-DT*. Several translation prediction algorithms demonstrated that *STX17-DT* has no coding ability(Fig. 2F). *STX17-DT* was predicted to localize mainly to the cytoplasm, and weakly to the nucleus and exosomes (Fig. 2G). The subcellular localization of *STX17-DT* was then validated through RNA FISH (Fig. 2H). To study whether *STX17-DT* is involved in clinical ccRCC progression, we analyzed the expression of *STX17-DT* in our SCU cohort, in which each ccRCC patient experienced relapse or metastasis and received axitinib treatment. Remarkably, prolonged PFS was observed in the low *STX17-DT* expression group, indicating that *STX17-DT* might play an important role in axitinib resistance in ccRCC (Fig. 2I and Supplementary Tables 1, 2). In summary, *STX17-DT* was upregulated in axitinib-resistant cells and associated with poor outcomes in ccRCC patients.

### *STX17-DT* enhanced axitinib resistance in RCC cells

**Fig. 3:**
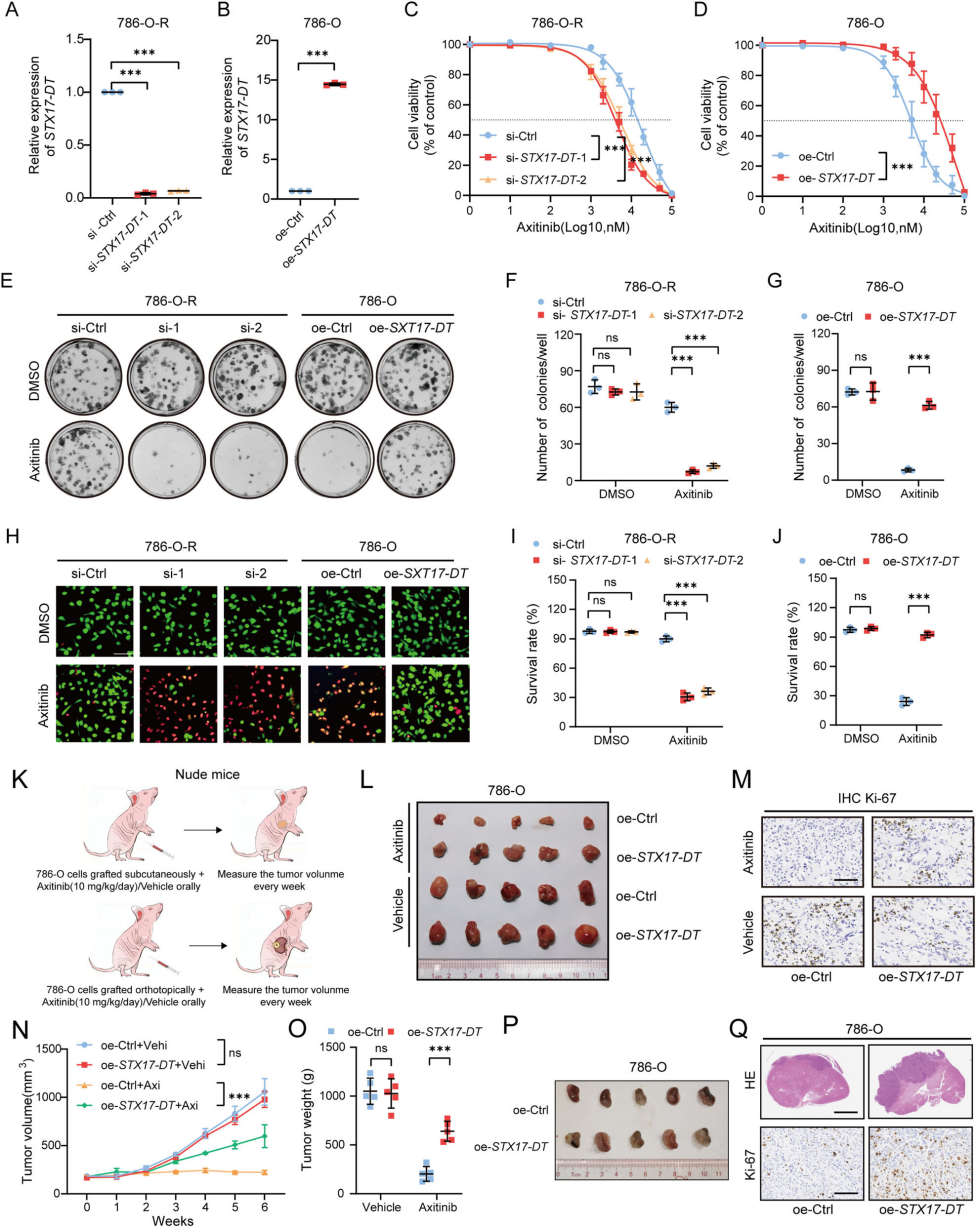
*STX17-DT* enhanced axitinib resistance in RCC cells. **A** Relative expression of *STX17-DT* in control and *STX17-DT*-knockdown cells. **B** Relative expression of *STX17-DT* in control and *STX17-DT* overexpressing cells. **C** Cell viability curves of control and *STX17-DT*-knockdown cells after treatment with axitinib. **D** Cell viability curves of control and *STX17-DT*-overexpressing cells after treatment with axitinib. **E** Colony formation assay of *STX17-DT*-knockdown and *STX17-DT*-overexpressing RCC cells after treated with axitinib. **F**, **G** Statistical plots of the number of clones formed. **H** Representative images of Calcein-AM staining of *STX17-DT-*knockdown and *STX17-DT*-overexpressing RCC cells after treated with axitinib. Scale bar, 100 μm. **I**, **J** Statistical plots of the survival rate. **K** Schematic illustration of the experimental flow in vivo. **L** Picture of the subcutaneous tumors of each group. **M** Representative images of Ki67 staining in each group. Scale bar, 100 μm. **N**, **O** Growth curves and weights of the subcutaneous tumors were measured. **P** Representative picture of orthotopic tumors. **Q** Representative images of H&E and Ki67 staining in each group. Scale bar for H&E, 2.5 mm. Scale bar for IHC, 100 μm. The data are shown as mean ± SD (error bars).

To explore the role of *STX17-DT* in axitinib resistance, we constructed *STX17-DT* knockdown and overexpression cell lines (Figs. 3A, B and S3A, S3B). We noted that resistance to axitinib was significantly attenuated in *STX17-DT* knockdown cells, with reduced IC_50_ values, decreased number of clones, and increased cell death after axitinib treatment. However, overexpression of *STX17-DT* showed the opposite results (Figs. 3C–J and S3C–3J).

We next examined the function of *STX17-DT* in vivo (Fig. 3K). The results revealed that *STX17-DT* overexpression was sufficient to confer tumor resistance to axitinib, leading to increased tumor sizes, heavier weights, and higher Ki-67 expression compared with those in the control group when treated with axitinib in vivo (Fig. 3L–O). Similar results were observed in the orthotopic model (Fig. 3P, Q).

### *STX17-DT* regulated ferroptosis by modulating the expression of IFI6

**Fig. 4:**
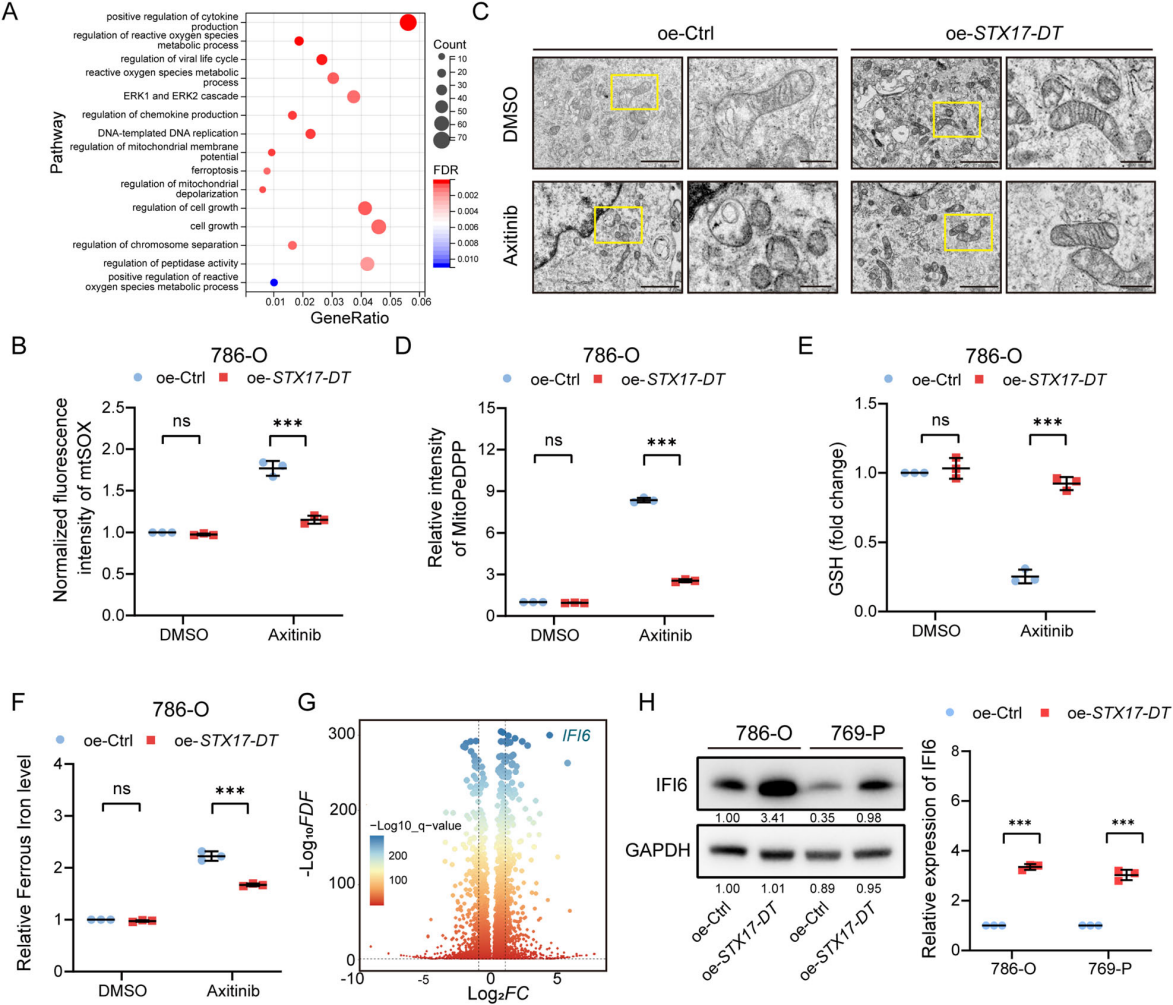
*STX17-DT* regulated ferroptosis by modulating the expression of IFI6. **A** GSEA of enriched pathways in *STX17-DT*-overexpressing 786-O cells. **B** Mitochondrial superoxide production was determined using the mitochondrial superoxide indicator. **C** The morphology of the mitochondria in the different groups was observed by electron microscopy. Left panel: scale bar, 2 μm. Right panel: scale bar, 500 nm. **D** The level of lipid peroxidation was assessed through MitoPeDPP intensity. **E** Relative levels of GSH in the different groups. **F** Relative level of ferrous iron in the different groups. **G** Volcano map showing genes that were differentially expressed after overexpressing *STX17-DT* in RCC cells. **H** Representative western blotting (left panel) and statistical analyses (right panel) of IFI6 protein expression levels in RCC cells overexpressing *STX17-DT*. The data are shown as mean ± SD (error bars).

To clarify the mechanism by which *STX17-DT* regulates axitinib resistance, we conducted transcriptome sequencing following *STX17-DT* overexpression. Differentially expressed genes were remarkably enriched in reactive oxygen species (ROS) metabolism, ferroptosis and pathways related to mitochondrial function (Fig. 4A). Mitochondrial superoxide levels were decreased in cells overexpressing *STX17-DT* after treated with axitinib, indicating that *STX17-DT* might protect RCC cells from axitinib-induced cell death through inhibiting reactive oxygen species and lipid peroxide accumulation in mitochondria (Figs. 4B and S4A). Considering that the ROS level is closely related to ferroptosis, we next explored the role of *STX17-DT* in ferroptosis. The overexpression of *STX17-DT* led to fewer abnormal mitochondrial morphologies induced by axitinib, such as swelling, disorganization and reduction or vanishing of cristae (Fig. 4C). Furthermore, exogenous *STX17-DT* led to changes in ferroptosis-related markers, including reduced levels of MitoPeDPP (mitochondrial lipid peroxide), increased production of GSH and decreased ferrous iron accumulation, indicating that *STX17-DT* might regulate axitinib resistance through ferroptosis (Figs. 4D–F and S4B–4D).

We subsequently explored the specific mechanism by which *STX17-DT* regulates mitochondrial function and ferroptosis. A volcano plot showed the differentially expressed genes after *STX17-DT* overexpression (Fig. 4G). Among them, interferon alpha inducible protein 6 (*IFI6*), an interferon-stimulated gene mainly enriched in the inner mitochondrial membrane, has been reported to exert function in ROS production [25, 26]. Therefore, we focused on the potential association between *STX17-DT* and *IFI6* level. The result showed that the protein level of IFI6 was increased in *STX17-DT*-overexpressing cells (Fig. 4H). However, the detailed mechanism needs further elucidation.

### *STX17-DT* specifically interacted with hnRNPA1

**Fig. 5:**
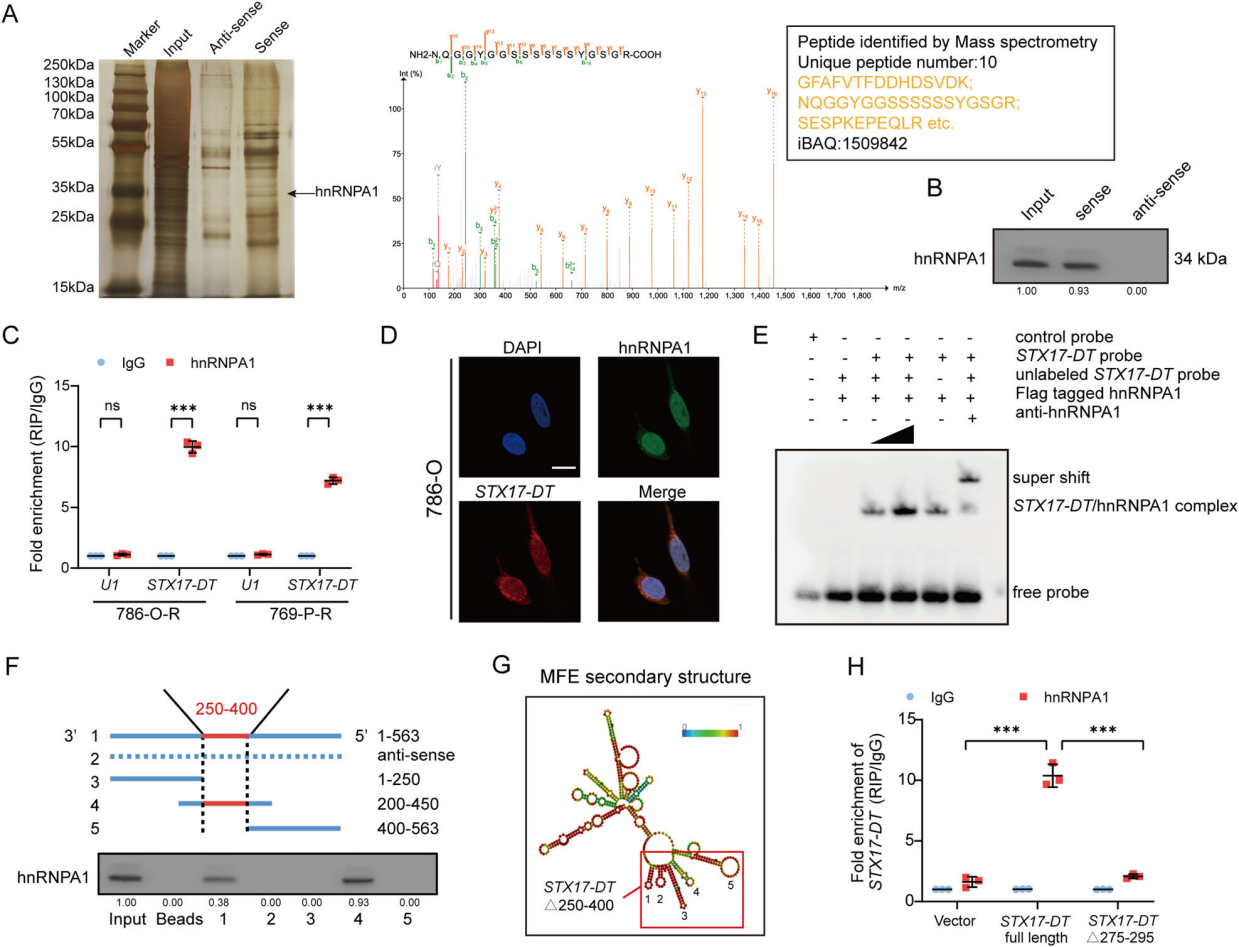
*STX17-DT* specifically interacted with hnRNPA1. **A** Left panel: SDS‒PAGE and silver staining of *STX17-DT-*bound proteins, hnRNPA1 is indicated by an arrowhead. Right panel: mass spectrometric profile of hnRNPA1. **B** The interaction between *STX17-DT* and hnRNPA1 was confirmed by Western blotting. **C** RIP was performed with anti-hnRNPA1 and control IgG antibodies, followed by qRT‒PCR. **D** Endogenous hnRNPA1 colocalized with *STX17-DT* in 786-O cells, as shown by confocal microscopy. Scale bar, 20 μm. **E** Biotin-labeled *STX17-DT* was incubated with flag-tagged hnRNPA1 protein, followed by RNA EMSA. Unlabeled probes were used for competitive binding. **F** Truncated *STX17-DT* probes were used in an RNA pulldown assay to identify the core region necessary for binding hnRNPA1. **G** The secondary structure of *STX17-DT* was predicted by RNAfold website (http://rna.tbi.univie.ac.at/cgi-bin/RNAWebSuite/RNAfold.cgi). The red box indicates the potential *STX17-DT* binding loop. **H** RIP assays after the overexpression of truncated *STX17-DT* in 786-O cells. The data are shown as mean ± SD (error bars).

To reveal the mechanisms by which *STX17-DT* regulates IFI6 expression, we performed an RNA pulldown assay with biotin-labeled *STX17-DT* probes followed by silver staining (Fig. 5A). The specific band was identified to be hnRNPA1 through mass spectrometry. hnRNPA1 is a member of the hnRNP subfamily and is known for its significant involvement in regulating mRNA synthesis, stability, translation, and extracellular vesicle packaging [27–29]. The interaction between *STX17-DT* and hnRNPA1 was further confirmed by western blotting after RNA pulldown and RNA immunoprecipitation (Fig. 5B, C). Confocal microscopy revealed that *STX17-DT* colocalized with hnRNPA1 in 786-O cells (Fig. 5D). An RNA electrical mobility shift assay (EMSA) also validated the specific binding of *STX17-DT* to hnRNPA1 (Fig. 5E). To further characterize the binding domain of *STX17-DT*, we constructed a series of *STX17-DT* truncations to perform an RNA pulldown assay. As indicated, the 250–400 nt of *STX17-DT* was necessary for binding with hnRNPA1 (Fig. 5F). The RNA secondary structure of *STX17-DT* was predicted *via* the RNA fold web server according to the minimum free energy (Fig. 5G). Several stem-loop structures were predicted at 250–400 nt of *STX17-DT*. We selectively deleted the 275–295 nt in the second loop of this region, which contains the binding sequence of hnRNPA1 (ATTTA) [30]. As anticipated, deletion of the linker region (275–295 nt) resulted in a diminished ability to bind with hnRNPA1 (Figs. 5H and S5A). In conclusion, *STX17-DT* might exert its function through specifically interacting with hnRNPA1.

### *STX17-DT* recruited hnRNPA1 to the 3’UTR of the *IFI6* gene

**Fig. 6:**
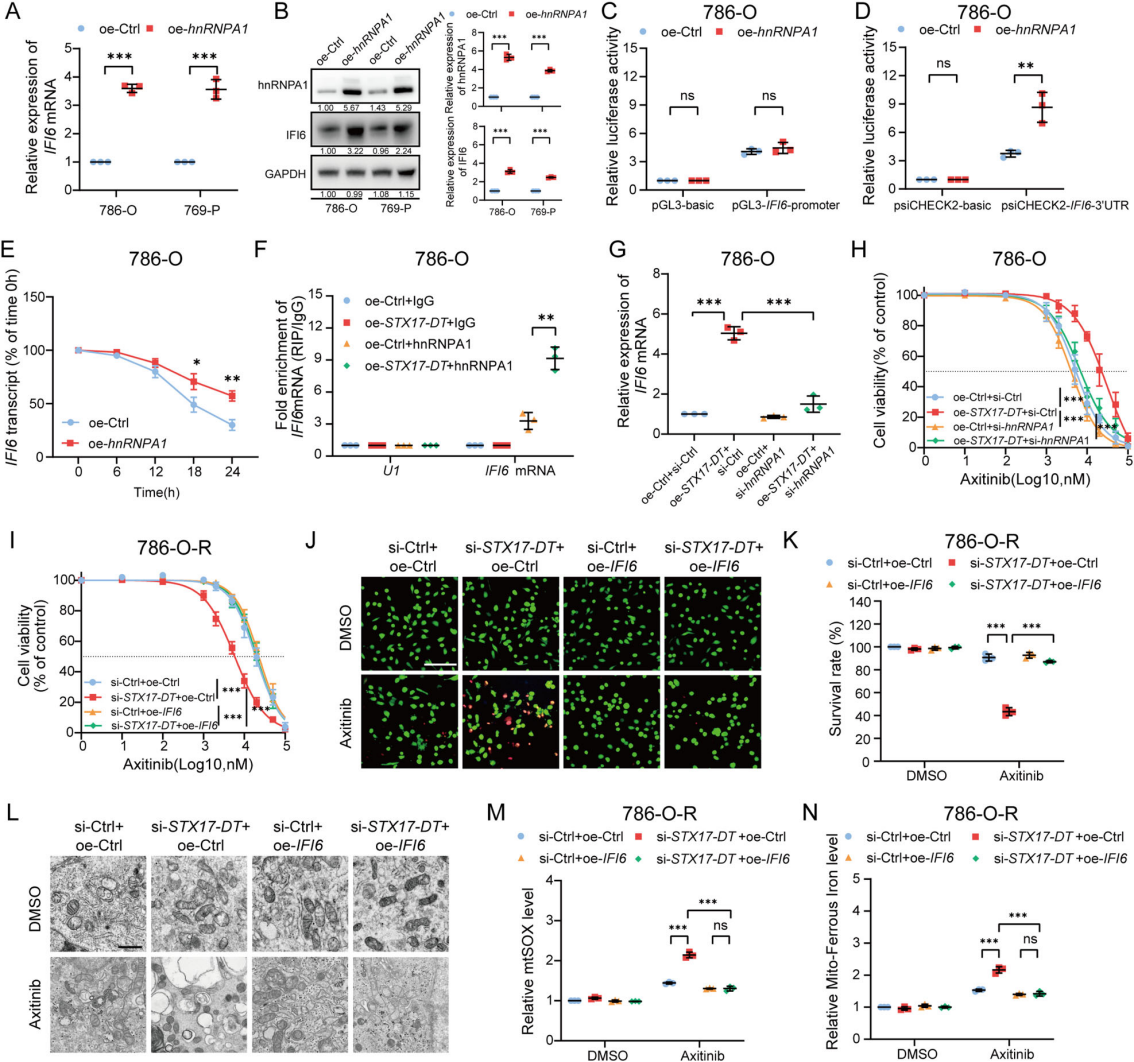
*STX17-DT* recruited hnRNPA1 to the 3’UTR of the *IFI6* gene. **A** Relative expression of *IFI6* mRNA in hnRNPA1-overexpressing RCC cells. **B** Representative western blotting (left panel) and statistical analyses (right panel) of hnRNPA1 protein and IFI6 protein expression levels in hnRNPA1-overexpressing RCC cells. **C** Relative luciferase activity of the *IFI6* promoter after hnRNPA1 overexpression. **D** Relative luciferase activity of the *IFI6* 3’UTR after hnRNPA1 overexpression. **E** RNA stability assay in hnRNPA1-overexpressing RCC cells. **F** Relative fold enrichment of *IFI6* mRNA in different groups according to the RIP assay. **G** Relative expression of *IFI6* mRNA in the different groups. **H** Silencing of hnRNPA1 suppressed axitinib resistance regulated by *STX17-DT* overexpression. Cell viability was measured by CCK-8 assay. **I***IFI6* overexpression rescued axitinib resistance in *STX17-DT*-knockdown RCC cells. **J** Calcein-AM staining was used to determine cell death after treatment with DMSO or axitinib. Scale bar, 100 μm. **K** Survival rates of the different groups. **L** The morphology of the mitochondria in the different groups was observed via electron microscopy. Scale bar, 2 μm. **M** Relative mtSOX levels in each group. **N** Relative level of ferrous iron in different groups. The data are shown as mean ± SD (error bars).

We next explored the role of the *STX17-DT-*hnRNPA1 interaction in the regulation of IFI6 expression. We found that both the mRNA and protein levels of IFI6 increased after hnRNPA1 overexpression (Fig. 6A, B). Further experiments revealed that hnRNPA1 enhanced the activity of the *IFI6* 3’UTR luciferase reporter, rather than the transcriptional activity of the *IFI6* promoter (Figs. 6C, D and S6A, S6B). The 3’UTR is known to be important for the structure and stability of RNA molecules, and we next validated the effect of hnRNPA1 on *IFI6* mRNA stability. RNA stability assays revealed that overexpression of hnRNPA1 decelerated the decay of *IFI6* mRNA, leading to increased RNA stability (Figs. 6E and S6C). Interestingly, *STX17-DT* overexpression increased the binding of hnRNPA1 to *IFI6* mRNA (Figs. 6F and S6D). Silencing hnRNPA1 rescued the *STX17-DT-*induced increase in *IFI6* mRNA (Figs. 6G and S6E). Downregulation of hnRNPA1 also impaired axitinib resistance mediated by *STX17-DT* overexpression (Figs. 6H and S6F). The above results showed that *STX17-DT* may recruit hnRNPA1 to the 3’UTR of the *IFI6* gene, thereby increasing the stability of *IFI6* mRNA.

To further evaluate the role of the *STX17-DT*/IFI6 axis in axitinib resistance, we performed rescue experiments. As expected, *IFI6* overexpression restored resistance to axitinib in *STX17-DT*-knockdown RCC cells (Figs. 6I and S6I, S6J, S6M). Calcein-AM staining for survival assessment also confirmed this observation (Figs. 6J, K and S6G, S6H, S6K, S6L, S6N, S6O). Mechanistically, exogenous overexpression of IFI6 suppressed ferroptosis induced by *STX17-DT* knockdown, as evidenced by the morphology of the mitochondria, decreased mtSOX and mito-ferrous iron levels (Figs. 6L–N and S6P, S6Q). Overall, *STX17-DT* enhanced axitinib resistance *via* hnRNPA1-mediated regulation of *IFI6* mRNA stability.

### *STX17-DT* transmitted axitinib resistance *via* hnRNPA1-mediated EV packaging

**Fig. 7:**
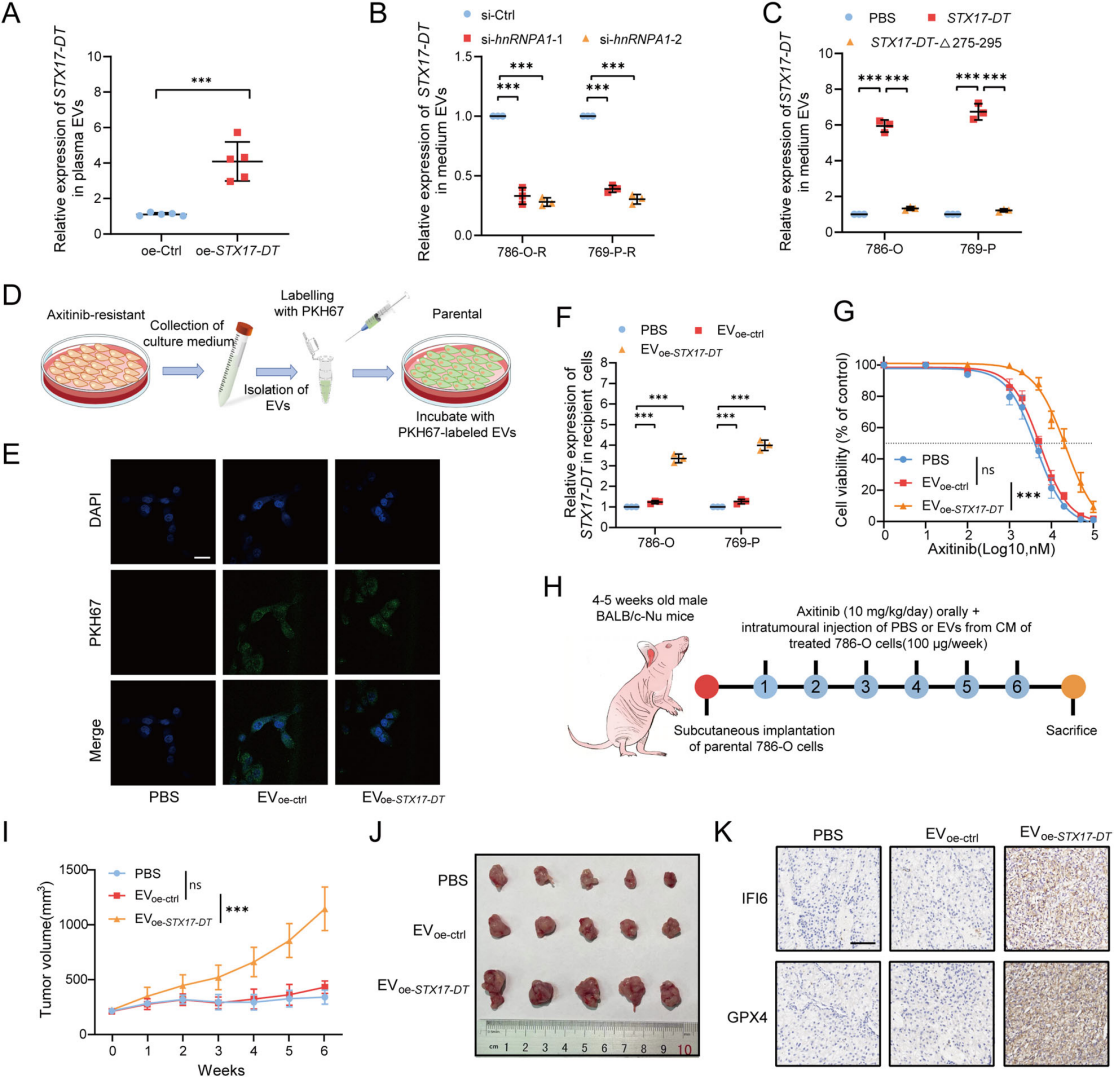
*STX17-DT* transmitted axitinib resistance *via* hnRNPA1-mediated EV packaging. **A** qRT‒PCR analysis of EV-packaged *STX17-DT* was collected from the plasma in the previous orthotopic experiment (Fig. 3P). **B** qRT‒PCR analysis of EV-packaged *STX17-DT* isolated from CM after silencing hnRNPA1. **C** qRT‒PCR analysis of EV-packaged *STX17-DT* released by cells overexpressing full-length or truncated *STX17-DT*. **D** Schematic diagram of EV labeling and internalization. **E** Representative confocal images of internalized EVs labeled with PKH67. Scale bar, 20 μm. **F** qRT‒PCR analysis of *STX17-DT* expression after incubation with the indicated EVs or PBS. **G** Cell viability assay of RCC cells incubated with the indicated EVs or PBS at the indicated concentrations of axitinib. **H** Schematic illustration of the establishment of the in vivo tumor model. **I** Growth curves of the subcutaneous tumors were generated. **J** Representative image of tumors after surgical resection. **K** Representative images of IFI6 and GPX4 staining in each group. Scale bar, 100 μm. The data are shown as mean ± SD (error bars).

Previous studies have indicated that noncoding RNAs can be packaged into extracellular vesicles and transmit drug resistance to other cancer cells [24, 31]. hnRNPA1 was also reported to mediate the localization of ncRNAs in EVs [29, 32]. Therefore, we suspected that hnRNPA1 might guide *STX17-DT* into EVs. We first extracted and validated the characteristics of the EVs (Fig. S7A–S7C). We found that the expression pattern of EV-packaged *STX17-DT* was similar to that of cellular *STX17-DT*, suggesting that *STX17-DT* might be packaged into EVs and exert its functions via EV transmission (Fig. S7D, S7E). Intriguingly, the expression of EV-packaged *STX17-DT* in plasma collected in the previous orthotopic experiment (Fig. 3P) exhibited a similar trend (Figs. 7A and S7F–S7H). Silencing hnRNPA1 inhibited the loading of *STX17-DT* into EVs (Fig. 7B). Further exploration revealed that *STX17-DT* truncation without the binding domain of hnRNPA1 was not enriched in EVs, which confirmed that *STX17-DT* could be packaged into EVs through binding with hnRNPA1 (Fig. 7C).

To identify the function of EV-packaged *STX17-DT*, we first confirmed the internalization of the EVs (Fig. 7D, E). EVs isolated from the cell medium(CM) of *STX17-DT*-overexpressing cells increased the axitinib resistance of recipient cells in vitro (Fig. 7F, G), corroborating the results obtained from EVs isolated from axitinib-resistant cells (Fig. S7I, S7J). In the xenograft model, tumors injected with EV_oe-*STX17-DT*_ exhibited a significantly faster growth rate and increased weight after axitinib treatment, with increased expression of IFI6 and GPX4 (Figs. 7H–K and S7K–S7M). Collectively, *STX17-DT* transmitted axitinib resistance *via* hnRNPA1-mediated EV packaging.

### Targeting *STX17-DT* restored axitinib sensitivity in vivo

**Fig. 8:**
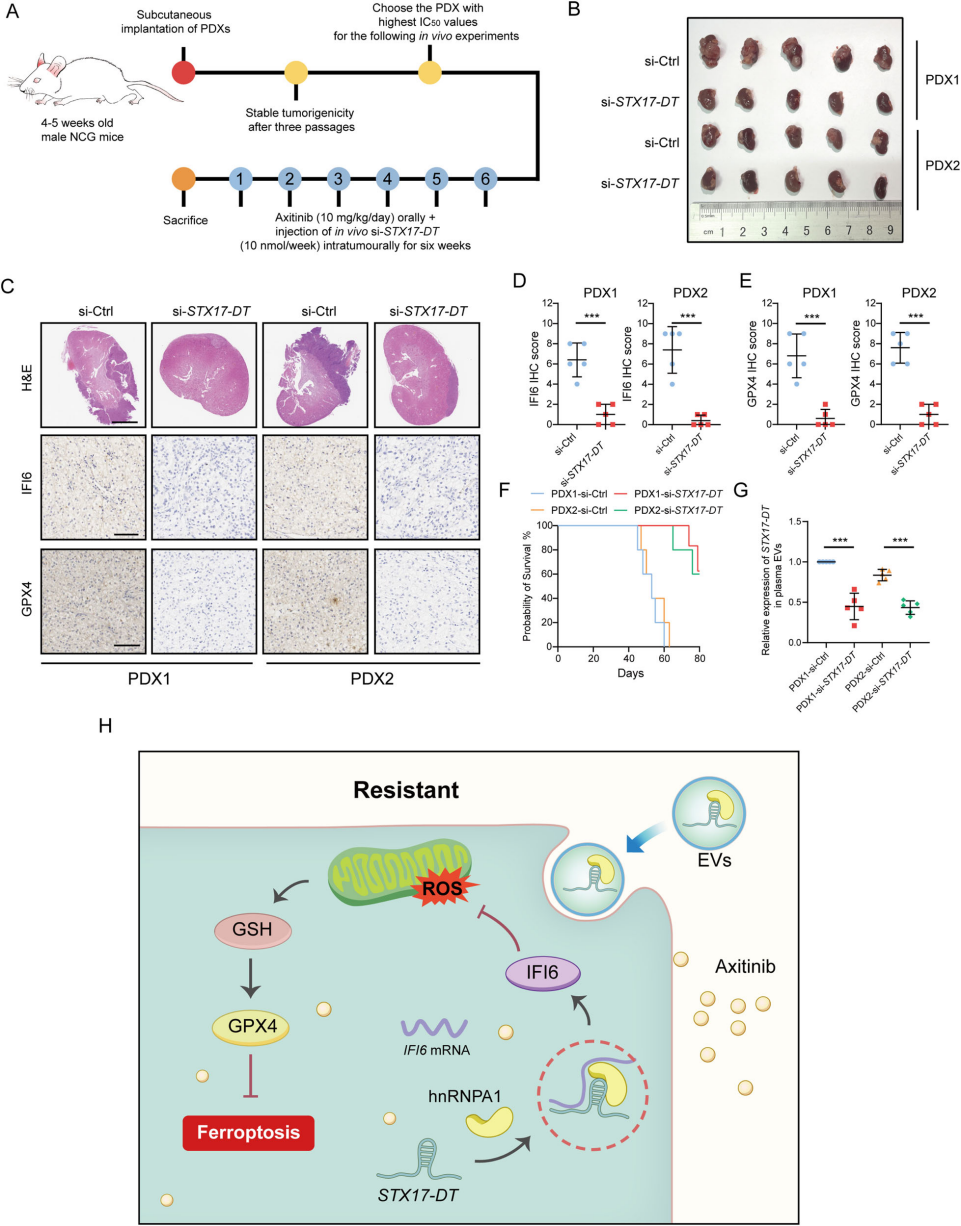
Targeting *STX17-DT* restored axitinib sensitivity in vivo. **A** Schematic illustration showing the establishment of the ccRCC PDX model and intratumoral injection of si-*STX17-DT* drugs. **B** Representative image of orthotopic tumors. **C** Representative images of H&E and IHC staining. Scale bar for H&E, 2.5 mm. Scale bar for IHC, 100 μm. **D**, **E** IHC scores of IFI6 and GPX4 in PDX1 and PDX2. **F** Kaplan‒Meier survival analysis of the four groups. **G** Relative expression of *STX17-DT* in plasma EVs. **H** Schematic illustration depicting the key findings of this study. The data are shown as mean ± SD (error bars).

Small interfering RNAs (siRNAs) are commonly used in biomedical research and clinical trials. Recently, siRNA treatment was demonstrated to be associated with significant sensitization to ferroptosis [33]. To verify the effect of targeting *STX17-DT* in combination with axitinib therapy in vivo, we generated five patient-derived tumor xenograft (PDX) models of ccRCC (Supplementary Table 3). Among these, the two PDXs with the most elevated *STX17-DT* expression in tumor tissue and the highest axitinib IC_50_ values were selected as candidates for siRNA administration (Figs. 8A and S8A, S8B). As shown in the results, the delay in tumor growth was considerably more pronounced in the combination group than in the single–axitinib–treated group, with reduced expression of IFI6 and GPX4 (Fig. 8B–E). Moreover, the combination treatment significantly prolonged the survival of NCG mice (Fig. 8F). Decreased *STX17-DT* expression in plasma EVs was detected after siRNA treatment (Fig. 8G). Overall, targeting *STX17-DT* augmented the antitumor effect of axitinib in vivo.

## Discussion

Although early-stage RCC is often cured by surgical resection, the prognosis for patients with metastatic RCC is very poor, with a 5-year survival rate of 8% [34]. In the last decade, there has been a major paradigm shift in the treatment of advanced RCC, and tumor angiogenesis has emerged as a significant therapeutic target [3, 4, 35]. Axitinib is an oral small-molecule TKI that selectively binds to and inhibits VEGF receptors, the clinical efficacy of which has been proven in metastatic RCC [7, 36]. However, almost all patients eventually develop disease progression because of drug resistance [37]. Therefore, new therapeutic approaches are urgently needed to overcome axitinib resistance and improve clinical outcomes. In this study, we first identified an axitinib resistance-related lncRNA *STX17-DT* and demonstrated its ability to promote axitinib tolerance. Mechanistically, *STX17-DT* stabilized *IFI6* mRNA through direct binding with hnRNPA1, thereby inhibiting axitinib-induced ferroptosis and promoting RCC cell survival. Our results suggest that targeting *STX17-DT* is a promising strategy for overcoming axitinib resistance in human RCC.

Cellular metabolism plays a vital role in the progression of renal cancer [38], with ferroptosis being a critical component. Ferroptosis is an iron- and lipid peroxidation-driven form of cell death that is distinct from necrosis, apoptosis, and autophagy. Numerous studies have demonstrated that modulating the ferroptotic cell death pathway can enhance the antitumor effects of drugs for cancer treatment [16, 39]. Some reports have shown that ferroptosis plays a vital role in resistance to targeted therapy resistance [40–42]. Considering that ferroptosis is a double-edged sword, there is still an urgent need to understand the mechanisms of ferroptosis regulation in RCC [43]. Kun et al. revealed that DPP9 overexpression suppressed ferroptosis and induced resistance to sorafenib in ccRCC cells, which was largely dependent on the NRF2 transcriptional target SLC7A11 [44]. Fatty acid oxidation mediated by malonyl-CoA decarboxylase repressed ccRCC progression through increasing ROS levels and inducing ferroptosis [45]. Because of their completely different mechanisms of action, targeting ferroptosis may represent a promising strategy to overcome the inefficiency of apoptosis-inducing drugs in cancer treatment [46]. In this study, we found that *STX17-DT* inhibited ferroptosis and conferred survival to neoplastic cells, especially when it was overexpressed in axitinib-resistant RCC cells. Cotreatment with *STX17-DT*-targeted compounds could be a potential option for monitoring resistance and increasing the therapeutic efficacy of axitinib in ccRCC.

The main feature of ferroptotic death is the iron-dependent accumulation of lipid-based ROS. In addition to its well-established role in virus infection, IFI6, an IFN-stimulated gene localized in the inner mitochondrial membrane, has been shown to play a pro-oncogenic role in various tumors and is associated with ROS accumulation. IFI6 depletion has been reported to suppress proliferation and induce apoptosis by increasing ROS accumulation in esophageal squamous cell carcinoma [47]. Knockdown of IFI6 enhanced the sensitivity to oxamate by enhancing the phosphorylation level of p38 and increasing ROS in CRC cells [26]. Our findings indicated that the expression of IFI6 is significantly upregulated in RCC cells overexpressing *STX17-DT*. We also proceeded to elucidate that *STX17-DT* inhibited ferroptosis through IFI6-mediated ROS clearance, thereby leading to the development of resistance to axitinib.

In recent years, lncRNAs have received attention as modulators of ferroptotic cancer cell death [48, 49]. Huang et al. reported that the *BDNF-AS*/WDR5/FBXW7 axis modulates ferroptosis in gastric cancer through the regulation of VDAC3 ubiquitination, thereby contributing to the progression of the disease [50]. *LINC00618* accelerated ferroptosis by increasing the levels of lipid ROS and iron, thereby attenuating the expression of lymphoid-specific helicases in leukemia [51]. Therapeutic strategies based on ferroptosis-related lncRNAs are expected to inspire new strategies for the treatment of ccRCC [52]. Here, we first revealed the function of *STX17-DT* in ferroptosis regulation, which is considered a potential target molecule for increasing the antitumor efficacy of axitinib in ccRCC.

A limitation of our study is that axitinib-resistant RCC cell lines could not reflect the microenvironment observed in humans during treatment. Ideally, the identification of axitinib-resistant-related biomarkers would be based on resistant tissue specimens or human PDX models. Second, additional plasma samples are needed to validate the drug resistance mechanism and explore the potential clinical prognostic value of EV-packaged *STX17-DT* identified in this study.

In summary, we constructed RCC cell lines with axitinib resistance via an in vivo model. We also provided the first detailed characterization of *STX17-DT* as an axitinib resistance-associated lncRNA. Mechanistically, *STX17-DT* promoted axitinib resistance through direct binding to hnRNPA1, thereby stabilizing *IFI6* mRNA and leading to ROS clearance and ferroptosis suppression. Our study demonstrated that *STX17-DT*-targeted therapy in conjunction with axitinib may overcome drug resistance and increase antitumor efficacy in ccRCC.

## Supplementary information

Supplelementary Data 1

Supplelementary Data 2

## Data Availability

All the data associated with this study are presented in the paper or in the Supplementary Data. Patient information, primers, and RNAi target sequences are presented in the Supplementary Data.

## Material availability

The materials supporting the findings of this study are available from the corresponding author upon reasonable request.
