# Supplementary material for: STX17-DT facilitates axitinib resistance in renal cell carcinoma by inhibiting mitochondrial ROS accumulation and ferroptosis
Source: Cell Death Dis. 2025 Feb 23;16(1):125. doi: 10.1038/s41419-025-07456-9 (PMC11847927; doi:10.1038/s41419-025-07456-9)
Supplement: Supplementary file 1 — Supplelementary Data 1 [file 41419_2025_7456_MOESM1_ESM.pdf]

Supplementary Data

Title: ***STX17-DT*** **facilitates axitinib resistance in renal cell carcinoma by inhibiting mitochondrial ROS accumulation and ferroptosis**

Author: *Yihui Pan*[1]†*, *Shuang Liu*[2]†, *Guannan Shu*[3]†, *Minyu Chen*[4]†, *Liangmin Fu*[4,6]†， *Cheng Chen*[1], *Yimeng Chen*[1], *Dong Xu*e[1]*, *and Xiaozhou He*[1]*

Affiliations:

[1] Department of Urology, the Third Affiliated Hospital of Soochow University, Changzhou 213000, China.

[2] Department of Oncology, the Third Affiliated Hospital of Soochow University, Changzhou 213000, China.

[3] Department of Urology, Guangzhou Women and Children's Medical Center, Guangzhou Medical University, Guangdong Provincial Clinical Research Center for Child Health; Department of Pediatric Surgery, Guangzhou Institute of Pediatrics, Guangdong Provincial Key Laboratory of Research in Structural Birth Defect Disease, Guangzhou Women and Children's Medical Center, Guangzhou Medical University, Guangzhou, 510623, Guangdong, China.

[4] Department of Urology, the Second Xiangya Hospital, Central South University, Changsha, Hunan 410000, China.

[5] Department of Urology, the First Affiliated Hospital, Sun Yat-sen University, Guangzhou 510080, China.

[6] Uro-Oncology Institute of Central South University, Changsha, Hunan, China.

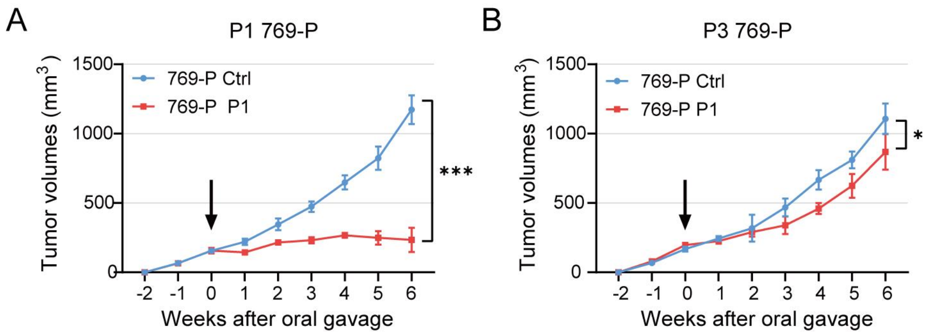

Figure S1. Construction of axitinib-resistant cell lines *in vivo*. (A,B) The construction of axitinib-resistant RCC cells through three successive passages. The arrow indicates the start of treatment. The data are shown as mean ± SD (error bars). * $p < 0.05$,*** $p < 0.001$.

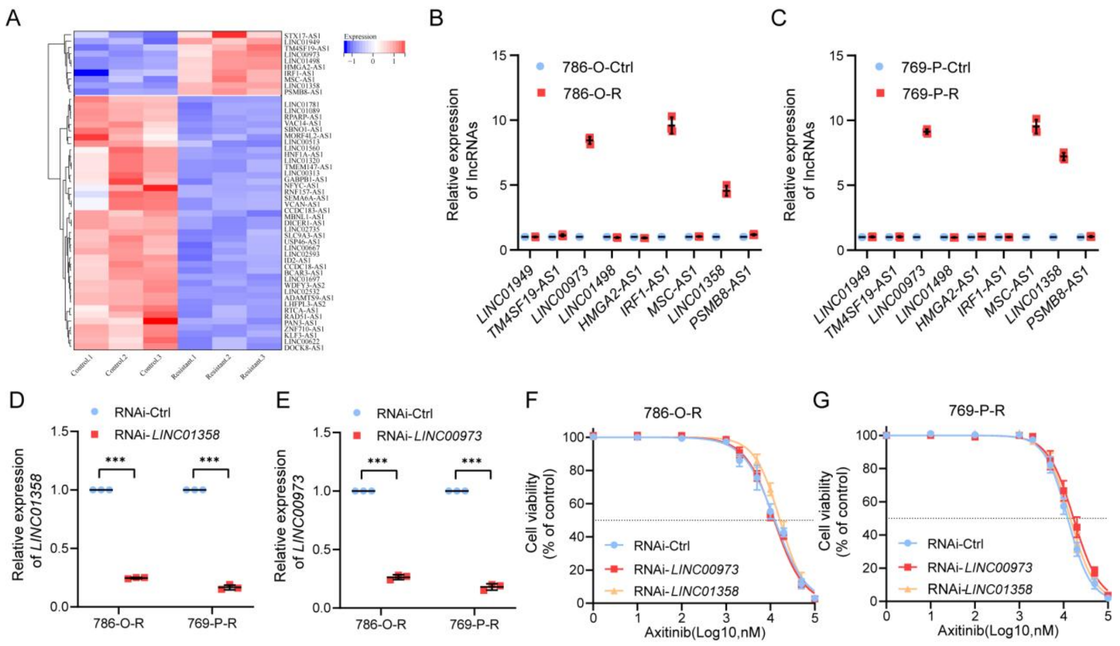

Figure S2. *STX17-DT* was upregulated in axitinib-resistant cells and associated with poor outcomes in ccRCC patients. (A) Heatmap of differentially expressed lncRNAs. (B,C) Relative expression of top 10 increased lncRNAs was validated by qRT-PCR in sensitive and resistant RCC cells. (D,E) Relative expression of *STX17-DT* was validated by qRT-PCR after silencing. (F) Cell viability assay in 786-O-R cells after silencing *LINC00973* and *LINC01358*. (G) Cell viability assay in 769-P-R cells after silencing *LINC00973* and *LINC01358*. The data are shown as mean ± SD (error bars). *** p <0.001.

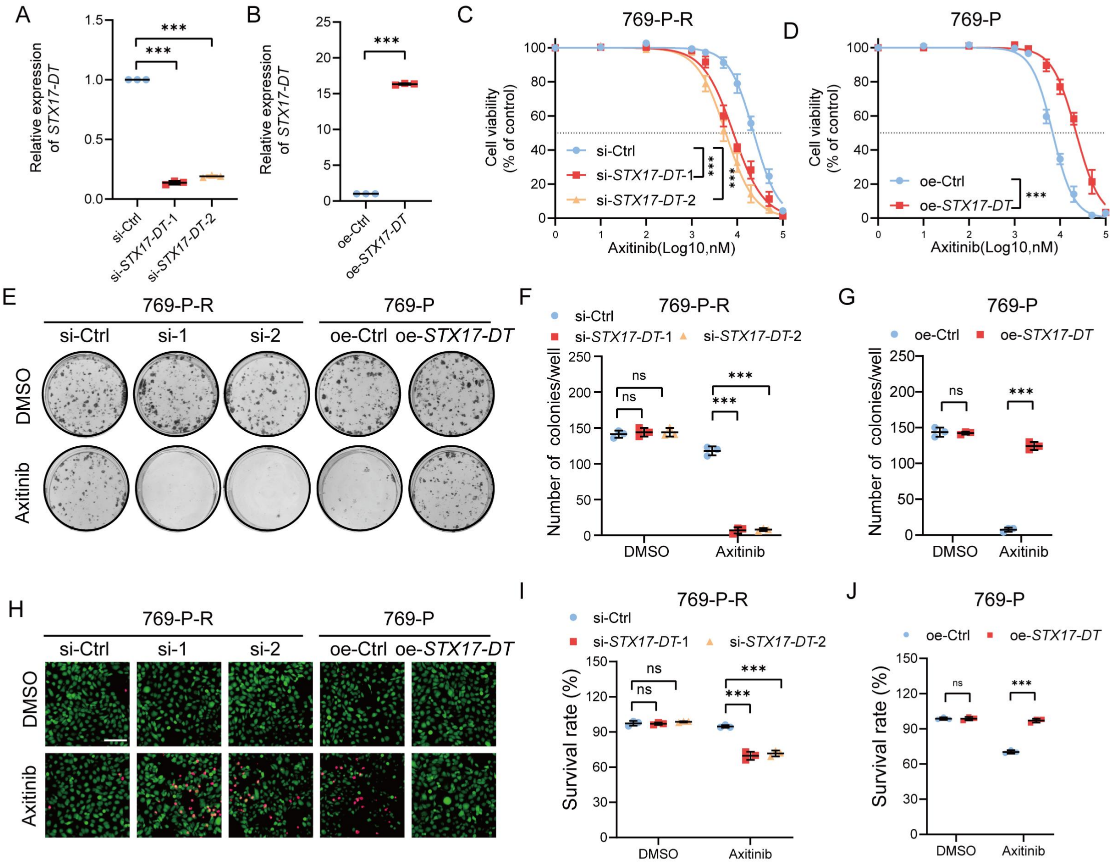

Figure S3. ***STX17-DT* enhanced axitinib resistance in RCC cells.** (A) Relative expression of *STX17-DT* in control and *STX17-DT* knockdown 769-P-R cells. (B) Relative expression of *STX17-DT* in control and *STX17-DT* over-expression 769-P cells. (C) Cell viability curve in control and *STX17-DT* knock down cells after treated with axitinib. (D) Cell viability curve in control and *STX17-DT* over-expression cells after treated with axitinib. (E) Colony formation assay of *STX17-DT* knock down and over-expression RCC cells after treated with axitinib. (F,G) The statistical plots of the number of clones formed. (H) Representative images of Calcein-AM staining of *STX17-DT* knock down and over-expression RCC cells after treated with axitinib. Scale bar, 100 μm. (I,J) The statistical plot of the survival rate. The data are shown as mean ± SD (error bars). *** $p < 0.001$.

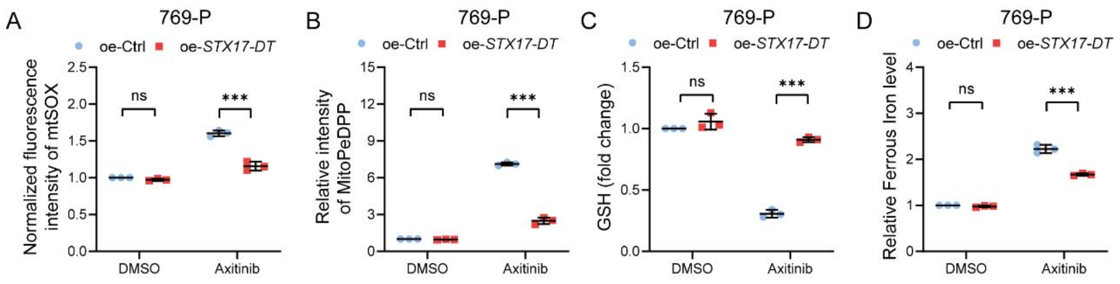

Figure S4. *STX17-DT* regulated ferroptosis by modulating the expression of IFI6. (A) Mitochondrial superoxide production was determined using the mitochondrial superoxide indicator. (B) The level of lipid peroxidation was assessed through MitoPeDPP intensity. (C) Relative level of GSH in different groups. (D) Relative level of ferrous iron in different groups. The data are shown as mean ± SD (error bars). *** p <0.001.

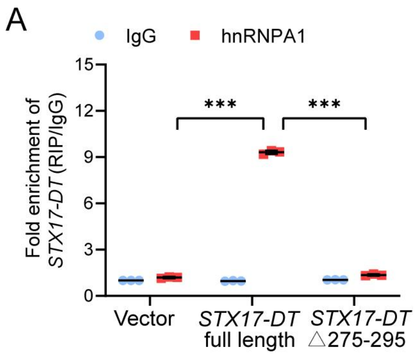

Figure S5. *STX17-DT* specifically interacted with hnRNPA1. (A) RIP was undertaken using anti-hnRNPA1 and control IgG antibodies, followed by qRT-PCR. The data are shown as mean ± SD (error bars). *** p <0.001.

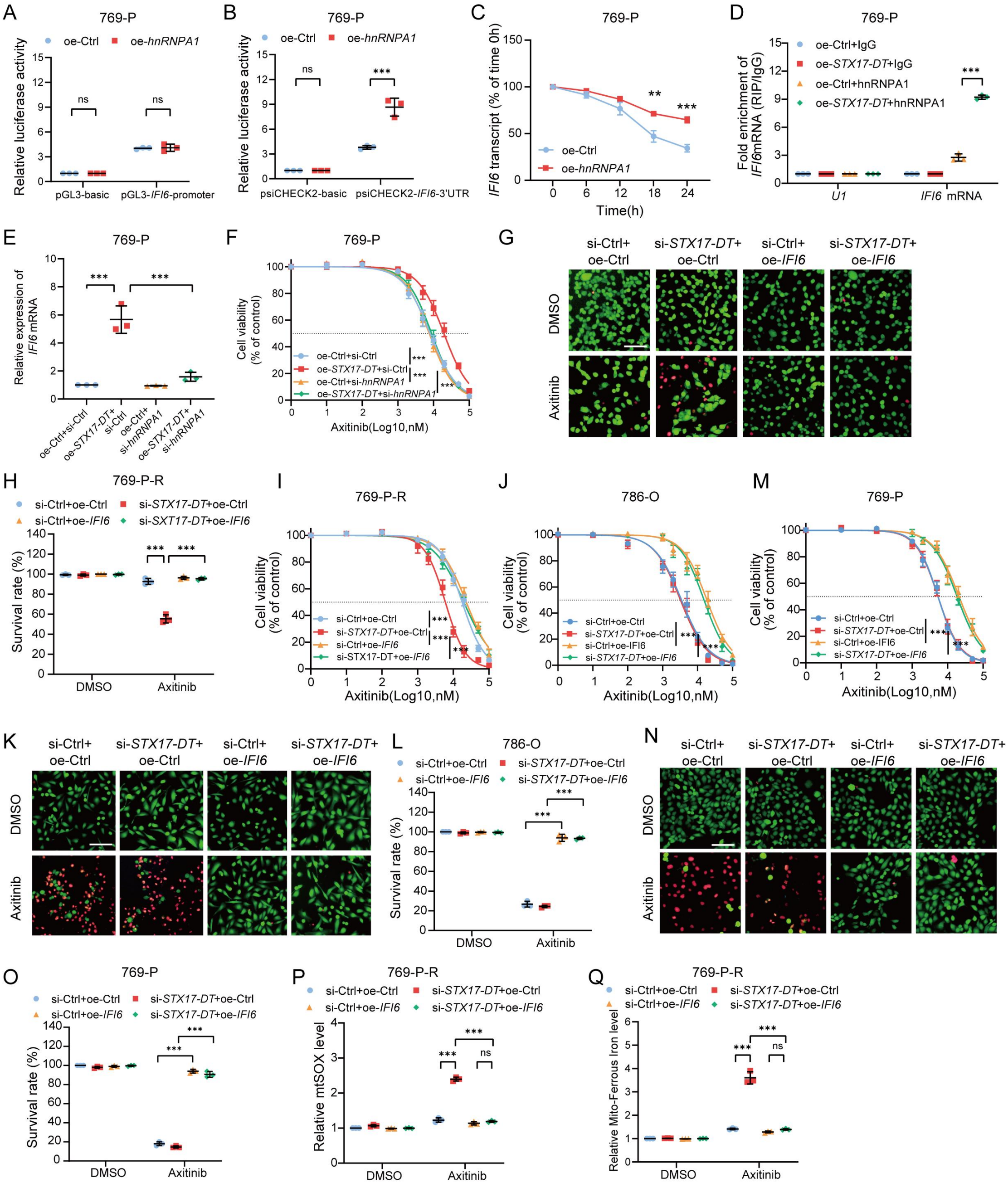

Figure S6. *STX17-DT* recruited hnRNPA1 to the 3'UTR of the *IFI6* gene. (A) Relative luciferase activity of *IFI6* promoter after hnRNPA1 overexpression. (B) Relative luciferase activity of *IFI6* 3'UTR region after hnRNPA1 overexpression. (C) RNA stability assay in hnRNPA1-overexpressed RCC cells. (D) Relative fold enrichment of *IFI6* mRNA in RIP experiment in different groups. (E) Relative expression of *IFI6* mRNA in different groups. (F) Silencing of hnRNPA1 suppressed

axitinib resistance regulated by *STX17-DT* overexpression. Cell viability was measured by CCK-8 assay. (G,K,N) Calcein-AM staining assay to determine cell death after treated with DMSO or axitinib. Scale bar, 100 µm. (H,L,O) Survival rate in different groups. (I, J, M) Overexpression of *IFI6* rescued axitinib resistance in *STX17-DT*-knockdown. (P) Relative mtSOX level in each group. (Q) Relative level of ferrous iron in different groups. The data are shown as mean ± SD (error bars). ** p <0.01, *** p <0.001.

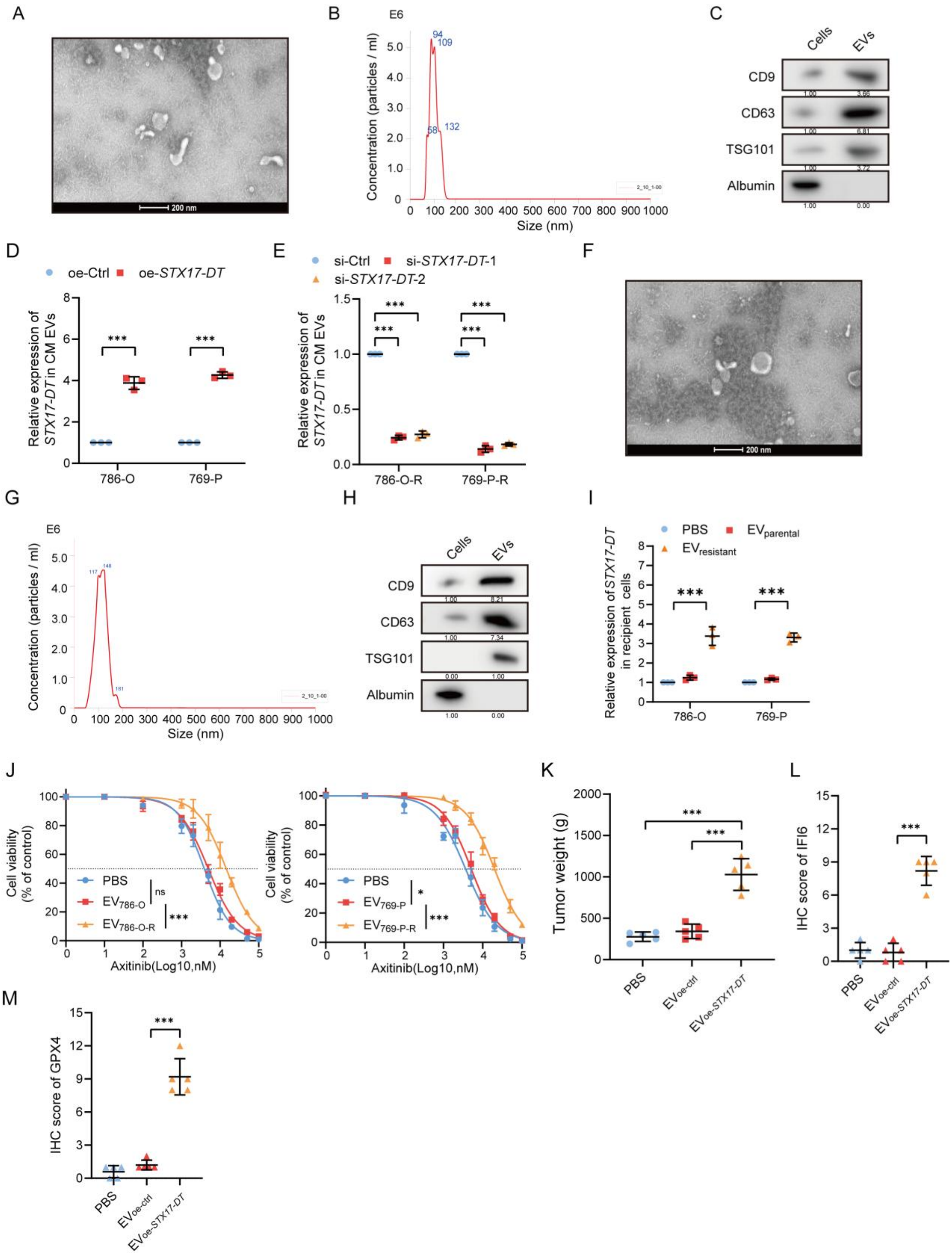

Figure S7. *STX17-DT* transmitted axitinib resistance *via* hnRNPA1-mediated EV packaging.

(A) Representative TEM images of CM-derived EVs. Scale bar: 200 nm. (B) Particle size analysis of CM-derived EVs documented by Nanosight ns300. (C) Western blotting analysis of protein markers of CM-derived EVs. (D,E) qRT-PCR analysis of EV-packaged *STX17-DT* expression

released by *STX17-DT* overexpression and knockdown RCC cells. (F) Representative TEM images of plasma-derived EVs. Scale bar: 200 nm. (G) Particle size analysis of plasma-derived EVs documented by Nanosight ns300. (H) Western blotting analysis of protein markers of plasma-derived EVs. (I) qRT–PCR analysis of *STX17-DT* expression after incubation with the indicated EVs or PBS. (J) Cell viability assay of RCC cells incubated with the indicated EVs or PBS at the indicated concentrations of axitinib. (K) The tumor weight was measured after surgical dissection (n=5). (L,M) IHC score of IFI6 and GPX4. The data are shown as mean ± SD (error bars). * p <0.05, *** p <0.001.

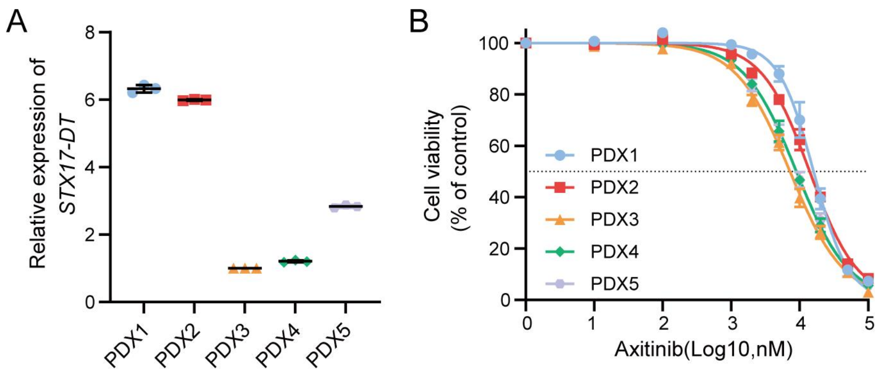

Figure S8. Targeting *STX17-DT* restored axitinib sensitivity *in vivo*. (A) qRT-PCR analysis of *STX17-DT* expressions in tumor tissues among PDX models. (B) PDX-derived RCC cells were treated with the indicated concentrations of axitinib for 60 h. Cell viability was calculated by the CCK8 assay. The data are shown as mean ± SD (error bars).

Supplementary Table 1. Correlations between *STX17-DT* tissue expressions and clinical characteristics of 42 ccRCC patients in the SCU Cohort.

Parameters	Number of cases	*STX17-DT* Low	*STX17-DT* High	*P* value
Total	42	21	21	
Age				0.346
≤ 60	17	7	10	
> 60	25	14	11	
Gender				0.469
male	32	17	15	
female	10	4	6	
WHO/ISUP Grade				1.000
I-II	16	8	8	
III-IV	26	13	13	
IMDC risk groups				0.739
Favorable& Intermediate	29	15	14	
Poor	13	6	7	

Supplementary Table 2. Uivariate and multivariate cox regression analysis of variables associated with PFS in the SCU cohort.

Parameters	Univariate analysis		Multivariate analysis	
	**HR (95%CI)**	***P* value**	**HR (95%CI)**	***P* value**
Age (＞60 years vs. ≤60 years)	0.92(0.45-1.87)	0.813	0.92(0.44-1.91)	0.817
Gender (male vs. female)	1.00(0.43-2.34)	0.994	0.73(0.28-1.90)	0.518
WHO/ISUP Grade (I-II vs. III-IV)	0.78(0.38-1.59)	0.490	0.65(0.29-1.45)	0.291
STX17-DT expression in tissues (low vs. high)	2.19(1.06-4.51)	0.033	2.62(1.20-5.75)	0.0016
IMDC risk groups (favorable & intermediate vs. poor)	1.98(0.92-4.26)	0.083	2.24(1.01-4.95)	0.047

HR=hazard ratio. CI= confidence interval.

Supplementary Table 3. Clinical characteristics of RCC patients in the PDX models.

Patient ID	Age	Gender	Pathological Diagnosis	Sample Type	TNM Stage	WHO/ISUP Grade	Tumor Thrombus	Treatment
1	66	Male	ccRCC	Primary	3	2	Yes	Nephrectomy
2	76	Female	ccRCC	Primary	3	3	Yes	Nephrectomy
3	59	Female	ccRCC	Primary	1	3	No	Nephrectomy
4	44	Male	ccRCC	Primary	3	3	No	Nephrectomy
5	73	Female	ccRCC	Primary	3	3	No	Nephrectomy

Supplementary Table 4. Targeted sequences of siRNA oligos and shRNAs used in this study.

Name	Targeted sequences
si-*STX17-DT*-1	TTCCTTGATAAGCTGTTTAAACC
si-*STX17-DT*-2	TCCAAATAAAGGCAATATAGTTT
ASO-h-*STX17-DT*	CAATATAGTTTCACTGGAA
si-*hnRNPA1*-1	CAGCTATGACAGCTATAACAACG
si-*hnRNPA1*-2	CGGAAACCTTGGTGTAGTTGAAC
si-*LINC01358*	TGGCTACATCACTGAAATGGAAT
ASO-h-*LINC01358*	CCAAGAACATACAAGAGAA
si-*LINC00973*	TTCTTTGATGAGCTAGAAACTGT
ASO-h-*LINC00973*	GGAATTGATTACAATTCTA

Supplementary Table 5. Primers used in this study for qRT-PCR analysis.

Primer names	Forward (5'-3')	Reverse (5'-3')
STX17-DT	ACCTGGTCAGGATGGACTCA	GCGAATCAGTTAATGTAGCAGCA
LINC01358	GCTCTCACTTATGGGGGCTT	AGGGAGAAGGGCCACTAGG
LINC00973	TACTGGGTCACCTCCAATGC	TCTCCCTCTGTGACCTCGGA
LINC01949	TGCAACAGTCTGGGAGCAAA	GCCAGGTCTGGCATACAGTT
TM4SF19-AS1	GCACAGGTCTGACTTCTCTGG	CCCCACCATGGGTTATGCTT
HMGA2-AS1	ACCTGTCACTCCTCCACTGA	GTTGACACCAGCTTGTTGGG
IRF1-AS1	AAAATGAAAGCGGTCGTGGC	TGGCCATCCTCAGGACTTGC
MSC-AS1	GACTGAGAGCCCAATGTCGT	AGGCCTCCTTTTTGCACCTT
LINC01498	GATCATGGCAAATGCCGGTC	GGACACCTCCTTGGGGAAAG
PSMB8-AS1	GGAAAGACATCGGACCGTCA	GAAACGTTGGTGTCCTTGGG
β-actin	CATCCGTAAAGACCTCTATGCCAAC	ATGGAGCCACCGATCCACA
U6	GCTTCGGCAGCACATATACTAAAAT	CGCTTCACGAATTTGCGTGTCAT
GAPDH	TGCACCACCAACTGCTTAGC	GGCATGGACTGTGGTCATGAG
U1	GGGAGATACCATGATCACGAAGGT	CCACAAATTATGCAGTCGAGTTTCCC

Supplementary Table 6. Primary antibodies applied in this study.

Antigens	Catalog	Manufacturer	Application
GAPDH	60004-1-Ig	Proteintech	1:10000 for WB
CD9 (D3H4P)	13403	Cell Signaling Technology	1:1000 for WB
CD63	A19023	ABclonal	1:1000 for WB
TSG101	A5789	ABclonal	1:1000 for WB
Albumin	4A1C11	Proteintech	1:5000 for WB
IFI6	A6157	ABclonal	1:1000 for WB
IFI6	KHC0430	Proteintech	4 drops with Primary Antibody for IHC
hnRNPA1	8443S	Cell Signaling Technology	1:100 for RIP
	A18094	ABclonal	1:1000 for WB
GPX4	59735	Cell Signaling Technology	1:2000 for IHC
