# Supplementary figures and images for: STX17-DT facilitates axitinib resistance in renal cell carcinoma by inhibiting mitochondrial ROS accumulation and ferroptosis
Source: Cell Death Dis. 2025 Feb 23;16(1):125. doi: 10.1038/s41419-025-07456-9 (PMC11847927; doi:10.1038/s41419-025-07456-9)

Figure 4

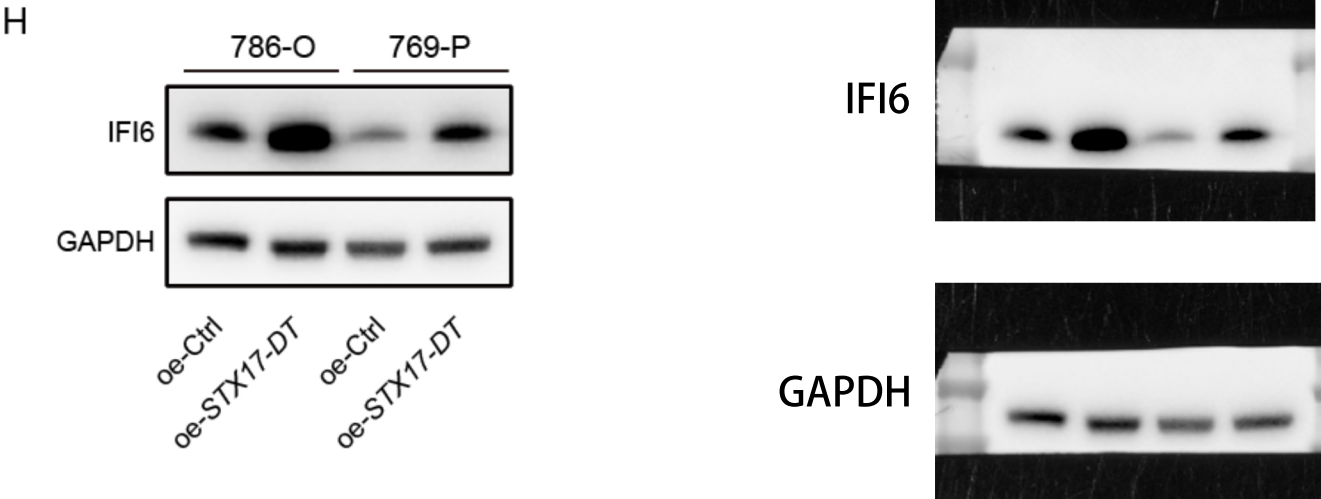

Figure 5

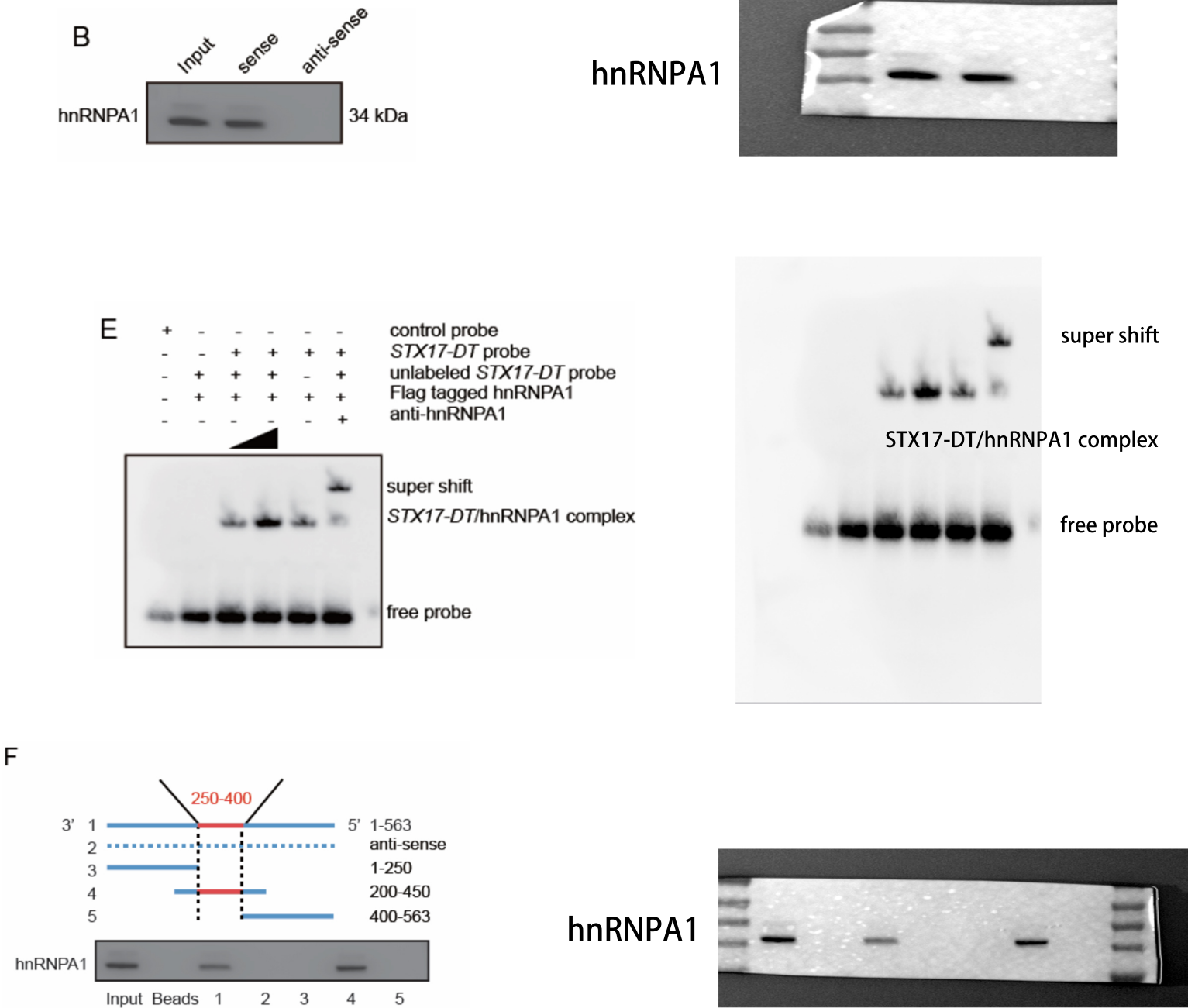

Figure 6

B

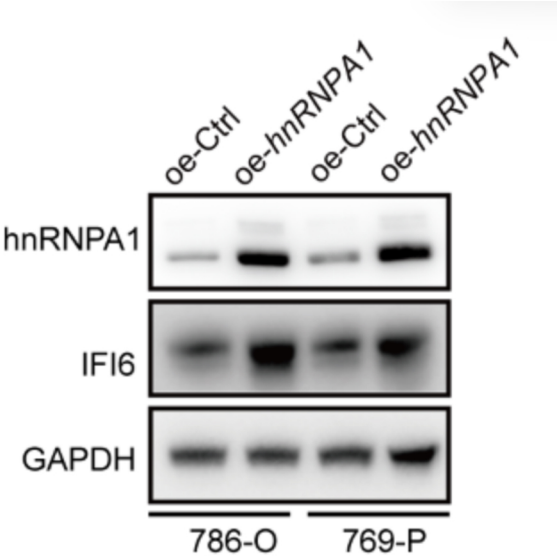

hnRNPA1

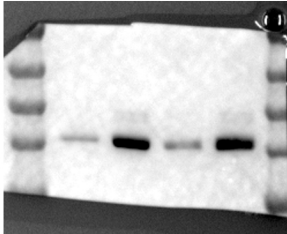

IFI6

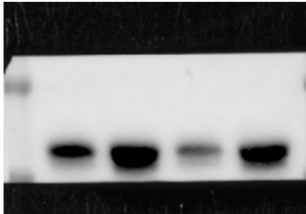

GAPDH

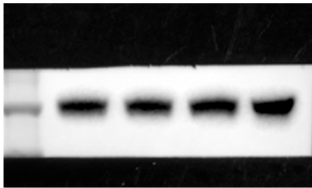

Figure S7

C

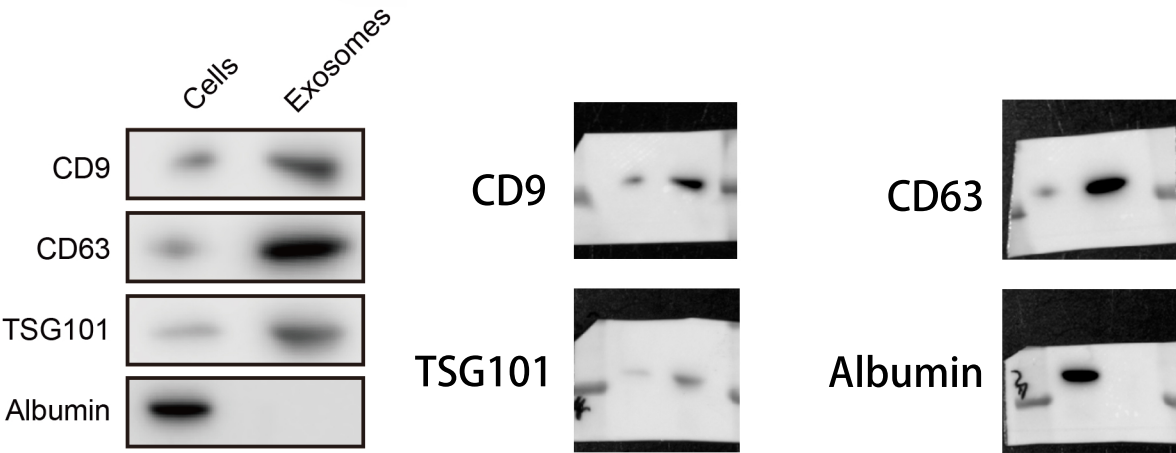

H

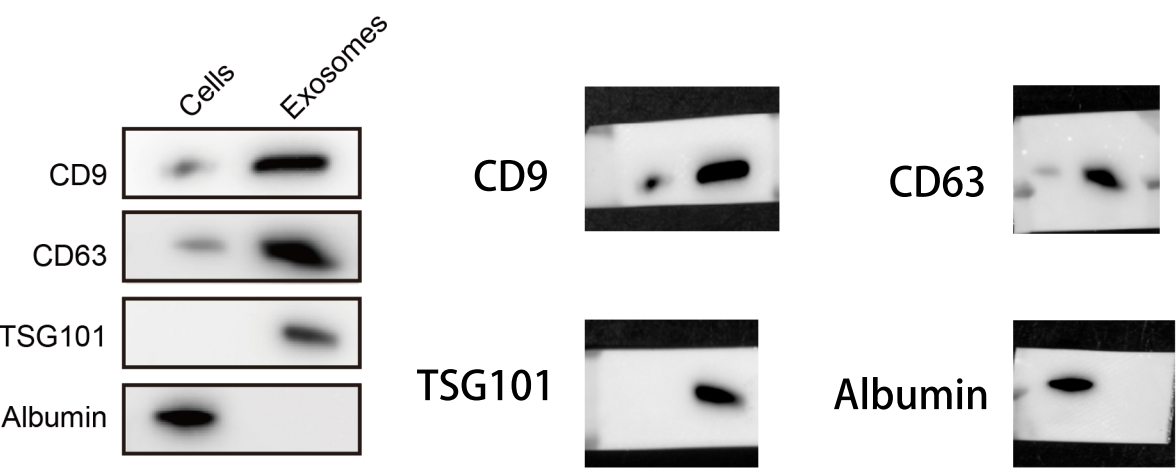

Supplement: Supplementary file 2 — Supplelementary Data 2 [file 41419_2025_7456_MOESM2_ESM.pdf]
